# Hepatic microRNAome reveals potential microRNA-mRNA pairs association with lipid metabolism in pigs

**DOI:** 10.5713/ajas.18.0438

**Published:** 2018-09-13

**Authors:** Jingge Liu, Caibo Ning, Bojiang Li, Rongyang Li, Wangjun Wu, Honglin Liu

**Affiliations:** 1 Department of Animal Genetics, Breeding and Reproduction, College of Animal Science and Technology, Nanjing Agricultural University, Nanjing, 210095, China

**Keywords:** Large White, Lipid Metabolism, Liver, MiRNA Expression Profiles

## Abstract

**Objective:**

As one of the most important metabolic organs, the liver plays vital roles in modulating the lipid metabolism. This study was to compare miRNA expression profiles of the Large White liver between two different developmental periods and to identify candidate miRNAs for lipid metabolism.

**Methods:**

Eight liver samples were collected from White Large of 70-day fetus (P70) and of 70-day piglets (D70) (with 4 biological repeats at each development period) to construct sRNA libraries. Then the eight prepared sRNA libraries were sequenced using Illumina next-generation sequencing technology on HiSeq 2500 platform.

**Results:**

As a result, we obtained 346 known and 187 novel miRNAs. Compared with the D70, 55 down- and 61 up-regulated miRNAs were shown to be significantly differentially expressed (DE). Gene ontology and Kyoto encyclopedia of genes and genomes enrichment analysis indicated that these DE miRNAs were mainly involved in growth, development and diverse metabolic processes. They were predicted to regulate lipid metabolism through adipocytokine signaling pathway, mitogen-activated protein kinase, AMP-activated protein kinase, cyclic adenosine monophosphate, phosphatidylinositol 3 kinase/protein kinase B, and Notch signaling pathway. The four most abundantly expressed miRNAs were miR-122, miR-26a and miR-30a-5p (miR-122 only in P70), which play important roles in lipid metabolism. Integration analysis (details of mRNAs sequencing data were shown in another unpublished paper) revealed that many target genes of the DE miRNAs (miR-181b, miR-145-5p, miR-199a-5p, and miR-98) might be critical regulators in lipid metabolic process, including acyl-CoA synthetase long chain family member 4, ATP-binding casette A4, and stearyl-CoA desaturase. Thus, these miRNAs were the promising candidates for lipid metabolism.

**Conclusion:**

Our study provides the main differences in the Large White at miRNA level between two different developmental stages. It supplies a valuable database for the further function and mechanism elucidation of miRNAs in porcine liver development and lipid metabolism.

## INTRODUCTION

The domestic pig is an important agricultural animal. Fat content is an important economic trait in pork production which is closely related to the pork flavor. Many researchers have tried their best to identify the key regulators which have important effects on pig fat content traits. As the largest digestive gland and one of the most important metabolic organs, the liver has the regulatory function of lipogenesis, fatty acid transportation and lipid metabolism [1]. Meanwhile, abnormal lipid metabolism can lead to non-alcoholic fatty liver in humans [2]. Intriguingly, the pig has a high similarity in anatomy and physiology with humans, which makes it emerge as a particularly valuable and suitable animal model for human pathologies and metabolism research. This shows that the study on the growth and development of liver tissue and lipid metabolism is greatly significant in animal production and human diseases.

The molecular mechanisms involved in liver lipid metab olism have been extensively and deeply studied. In recent years, a great number of studies have shown that microRNAs (miRNAs or miRs) play important roles in regulating liver lipid metabolism. For example, miR-122 which is abundantly expressed in liver, is the first validated miRNA that modulates fatty acids and cholesterol synthesis in liver by repressing aldolase-A,3-hydroxy-3-methylglutary-coenzyme A reductase (*HMGCoAR*) and AMP-activated protein kinase (*AMPK*) expression [3]. Previous studies showed that deletion of miR-122 could lead to decrease of serum triglyceride (TG) and cholesterol levels [4]. MiR-33a/33b were found to mediate many key genes which were involved in fatty-acid, cholesterol biosynthesis and intake, phospholipids and TG productions, by directly regulating sterol regulatory element binding protein (*SREBP*) expression [5]. SREBPs were considered to be key transcriptional regulators in lipid metabolism. Thus, studies on lipid metabolism-related miRNAs and their mechanisms in porcine liver would be meaningful.

MiRNAs are highly conserved endogenous non-coding single-stranded RNAs of about 22 nucleotides, which are able to regulate gene expression at the post-transcriptional level mainly by binding to the 3′-untranslated regions of target mRNAs and thereby inducing their degradation or repressing their translation [6]. Mounting evidences have indicated that they play significant roles in diverse biological processes, such as cell proliferation, differentiation, stress response and metabolism [7]. It indicates the importance of investigating the specific miRNAome of different developmental stages pigs.

The next-generation sequencing technology provides us a powerful research platform for miRNAome analysis. It could provide a large amount of data, and could be useful to predict the roles of individual miRNAs and their pathways. Nowadays, the miRNAomes of different mammal tissues have been revealed by the next-generation sequencing, including mice [8], cattle [9], and pig [10]. However, the study of miRNAome in Large White livers of different developmental stages is still lacking.

To better explore the roles and the underlying molecular mechanisms of candidate miRNAs in porcine liver lipid metabolism, we totally constructed eight miRNAs libraries from Large White livers of two different developmental stages, 70-day-old fetus (P70) and 70-day-old piglets (D70), and carried out miRNAome sequencing (RNA-Seq) to systematically explore the miRNAs associated with liver lipid metabolism, and to provide new theoretical basis for the regulation of liver lipid metabolism. This study could provide a valuable database for the further function and mechanism elucidation of the miRNAs roles in porcine liver lipid metabolism.

## MATERIALS AND METHODS

### Ethics statement

All experiments were conducted according to the guidelines of the Administration of Affairs Concering Experimental Animals and were approved by Animal Ethics Committee of Nanjing Agricultural University, Nanjing, Jiangsu, China.

### Sample selection and RNA isolation

Eight liver samples were randomly collected from White Large of P70 and of D70, with 4 biologocal repeats at each development period (the samples in each developmental period were full siblings). All the pigs were raised under the same environment with *ad libitum* water and food. All the samples were aseptically excised and stored at −80°C immediately, waiting for subsequent analysis.

In accordance with the manufacturer’s standard proto cols, total liver RNA was isolated using Trizol reagent Kit (Invitrogen, Carlsbad, CA, USA). The purity and concentration were analyzed by OD260/280 readings using NanoDrop 2000 (Thermo Scientific, Boston, MA, USA), and integrity was determined by capillary electrophoresis using RNA 6000 Nano assay Kit and the Agilent 2100 Bioanalyzer (Agilent Technologies, Santa Clara, CA, USA). Small RNA, of which 28S/18S ratios ranges from 1.8 to 2.0 and RNA integrity values between 8.0 and 10.0, was selected for further analysis.

### RNA library construction and sequencing

We constructed eight miRNAs libraries (four from livers of P70 and the other four from D70 individuals respectively) according to the vendor’s instructions of Illumina’s sample preparation and sequencing protocols in the Illumina HiSeq 2500 system (Illumina, San Diego, CA, USA), with the following steps: i) use 15% polyacrylamide gel electrophoresis (PAGE) to isolate 15 to 40 nt fragments. ii) add proprietary adapters to 5′and 3′end of the RNA which was isolated in step 1 respectively. iii) employ TruSeq Small RNA library Preparation Kit to reverse RNAs to cDNA by the real-time polymerase chain reaction (RT-PCR). iv) use 6% PAGE to purify the reversed cDNA. v) Then the eight prepared miRNA libraries were sequenced at Annorode Gene Technology Co., Ltd (Beijing, China), which generated more than 25 M (million) reads per library.

### Data analysis

Raw data were generated by the Illumina Genome Analyzer at Annorode-Beijing, China. Clean reads were obtained by removing the reads with adapters contamination, poly-N >10%, 3′ - adapters not found, and without the insert and length <15 and >35. Then clean reads were aligned to the porcine genome sequence (sscrofa 10.2, 2011) and miRbase database (release 22) using Bowtie (V1.1.2) [11] with the following steps: i) use public database NCBI and miRbase 22 to map the clean reads to the porcine and other mammals known miRNAs to get conserved known miRNAs; ii) use public database NCBI and Rfam to annotate the remaining other small RNAs, such as transfer RNA (tRNA), ribosomal RNA (rRNA) and small nucleolar RNA (snoRNA) et al., iii) use miRDeep 2 (V 0.2) (http://sourceforge.net/projects/mireap/) to predict the hairpin RNA structures of potentially novel microRNAs which did not map to miRbase 22.

### Identification and target prediction of the differentially expressed miRNAs

We used DEseq to analyze the differentially expressed (DE) miRNAs between porcine livers of P70 and D70. In order to characterize DE miRNAs, first, we used RPM values to compare miRNA expression levels between P70 and D70; then, corrected p-values (padj value) were employed to represent the significances of DE miRNAs based on Poisson distribution. We considered the miRNAs with threshold values of ≥2 absolute fold change and padj value of ≤0.05 as the DE miRNAs. In order to understand the functional roles what the DE miRNAs may play in porcine liver, we used miRanda (http://www.microrna.org/microrna/home.do), TargetScan (http://www.targetscan.org/vert_71/) and TarBase (http://diana.cslab.ece.ntua.gr/tarbase/) to predict their targeted genes. We considered the overlapping targeted genes as the final results.

### Functional annotation and pathways enrichment analysis

We performed hierarchical clustering algorithm to cluster the DE miRNAs. The target genes of the above DE miRNAs were submitted to gene ontology (GO) (http://www.geneontology.org) database for enrichment analysis. Then, we used Kyoto encyclopedia of genes and genomes (KEGG) project (http://www.genome.jp/kegg/pathway.html) to perform pathway analysis.

### qRT-PCR validation of the sequencing data, the lipid metabolism-associated DE miRNAs and their targeted genes

Quantitative reverse-transcription polymerase chain reaction (qRT-PCR) was used to validate the ten randomly selected DE miRNAs, the four lipid metabolism-associated DE miRNAs and their targeted genes respectively. Samples used for total RNA isolation were as the same as the samples used for miRNA sequencing. The complementary DNA (cDNA) used for validation of miRNAs was synthesized using the Mir-X microRNAs First Strand Synthesis Kit (TaKaRa, Dalian, China). Meanwhile, Primescript RT Master Kit (Takara, Dalian, China) was employed to generate the cDNA which was used for validation of the targeted genes. Primers used here were listed in Table 1. The qRT-PCR was performed with the SYBR Green Master Mix (Vazyme, Nanjing, China) and ABI StepOne Software v2.0 (Foster, CA, USA) using the following procedure: 95°C for 5 mins followed by 40 cycles of 95°C for 10 s, 60°C for 30 s, and then one cycle of 60°C for 1 min, 95°C for 15 s. Each assay was carried out on four biological replicates and each sample was performed in triplicates. U6 and the glyceraldehyde-3-phosphate dehydrogenase were used as the internal controls for the validation of miRNAs and targeted genes respectively. The relative expression level was measured using comparative 2^−ΔΔCt^ method [12].

### Statistical analysis

All assays were conducted on three biological replicates and each sample was run in triplicates. The results were presented as average±standard deviation. All the data were analyzed using SPSS v16.0 software (SPSS Inc., Chicago, IL, USA). T-test and one-way analysis of variance were used to analyze the significance of statistics. Less than 0.05 p value was considered to be statistically significant.

## RESULTS

### Summary of high-throughput sequencing data

**Figure 1:**
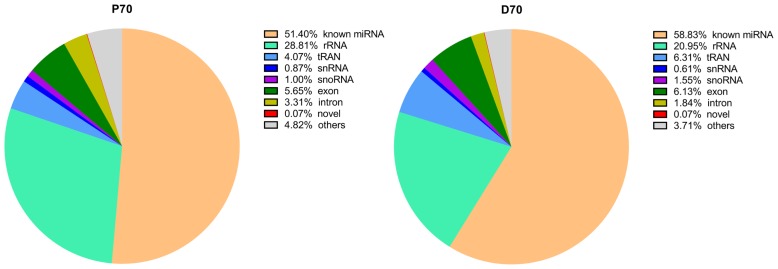
Annotation of small RNA sequences. P70, 70-day-old Large White fetus; D70, 70-day-old Large White piglets. Starting from the largest area, clockwisely all the areas represent known miRNA, rRNA, tRNA, snRNA, snoRNA, exon, intron, novel and others in turn.

**Figure 2:**
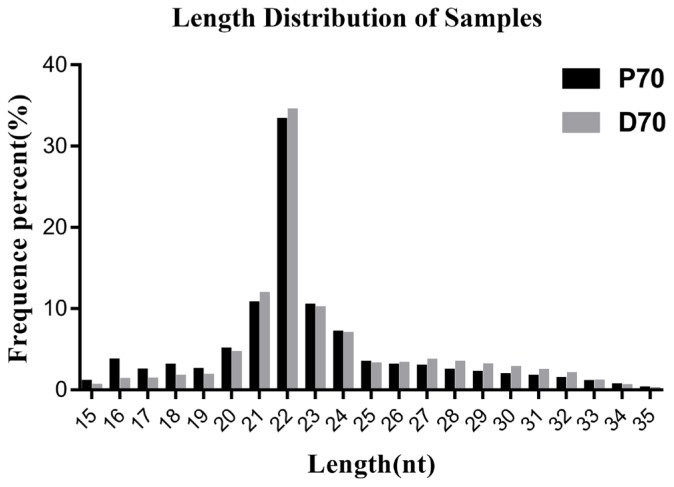
Length distribution of small miRNAs. P70, 70-day-old Large White fetus; D70, 70-day-old Large White piglets.

In current study, we totally constructed eight small RNA libraries using porcine livers at two different developmental stages (70-day-old fetus, P70.70-day-old piglets, D70). The eight libraries totally yielded 114,075,887 (P70) and 107,352,916 (D70) raw reads respectively. Totally, 90,625,206 (P70) and 88,964,163 (D70) clean reads were obtained for subsequent analysis. All the data were presented in Table 2. There was an average of 14,699,648 (P70) and of 14,625,727 reads (D70) which could be perfectly mapped to porcine genome sequence. An average of 7,555,649 (P70) and of 8,604,646 reads (D70) were annotated in miRbase 22.0 (sscrofa). Meanwhile, classification of these small RNA showed that an average of 51.4% (P70) and of 58.8% (D70) were known miRNAs, followed by rRNA and tRNA (Figure 1). Subsequently, we constructed a small RNA size distribution profile for each tissue. The size distribution was similar in all these libraries: the vast majority of small RNAs were 22 nt size, averagely acounting for 33.3% (P70) and 34.4% (D70) respectively (Figure 2), followed by 21, 23, and 24 nucleotides, which matched the length characteristics of mature miRNAs.

### Identification of the liver DE miRNAs

**Figure 3:**
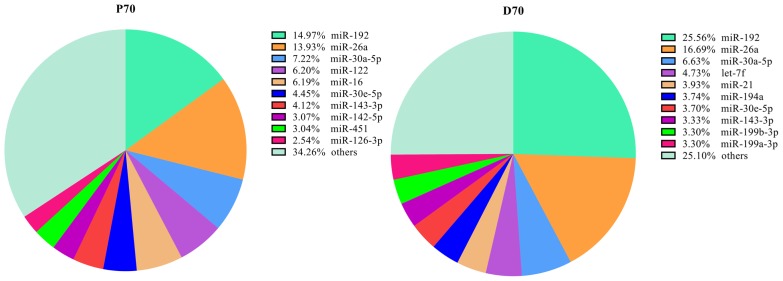
The ten top most abundantly known miRNAs in the livers of P70 and D70. P70, 70-day-old Large White fetus; D70, 70-day-old Large White piglets. Starting from the top right corner, clockwisely all the areas represent miR-192, miR-26a, miR-30a-5p, miR-122 (P70)/let-7f (D70), miR-16 (P70)/miR-21 (D70), miR-30e-5p (P70)/miR-194a (D70), miR-143-3p (P70)/miR-30e-5p (D70), miR-142-5p (P70)/miR-143-3p (D70), miR-451 (P70)/miR-199b-3p (D70), miR-126-3p (P70)/miR-199a-3p (D70) and others in turn.

**Figure 4:**
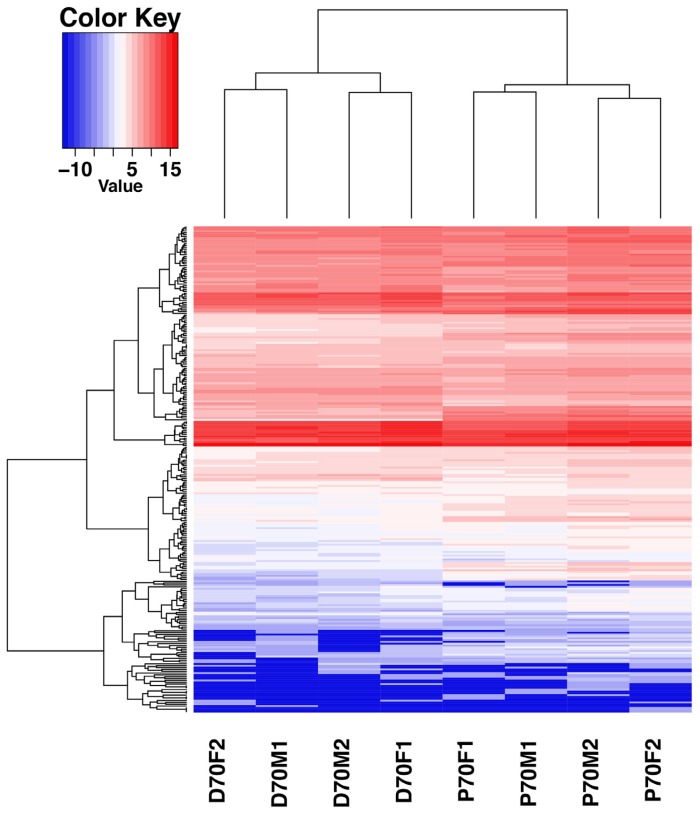
Hierarchical cluster analysis of DE miRNAs among the eight libraries. Porcine liver samples were clustered in accordance with the expression profiles of 116 differentially expressed miRNAs between P70 and D70. The sample clustering tree was shown at the bottom, and the miRNA clustering tree was reported on the left. The top color scale represents the relative expression level of the DE miRNAs. Red, high expression. Blue, low expression. P70F1, P70F2, P70M1, and P70M2, the four individuals of P70; D70F1, D70F2, D70M1, and D70M2, the four individuals of D70. DE, differentially expressed.

To identify the DE miRNAs which may play important roles in porcine livers, we compared the expression patterns of the hepatic miRNAs at P70 and D70. Totally as shown in Supplementary Table S1, Supplementary excel S1, and Supplementary excel S2, 346 mature known miRNAs corresponding to 279 precursor-miRNAs (pre-miRNA) and 187 novel miRNAs corresponding to 183 pre-miRNAs were identified. The ten most abundantly expressed known miRNAs in each period were listed in Figure 3, and nearly accounted for 65.7% and 74.9% of the known miRNAs in the P70 and D70 respectively. It indicates that these miRNAs may play fundamental roles in the porcine liver. Among the identified known miRNAs, 116 were differentially expressed, of which 61 were up-regulated, and 55 were down-regulated (D70 as the control, Supplemenary Table S1, Supplemenary excel S3). All the novel miRNAs showed no significant difference between P70 and D70 (Supplemenary excel S2). The relative expression levels of the majority novel miRNAs were very low and most were detected in only one of the eight libraries. Hierarchical cluster analysis of DE miRNAs showed that the four biological replicates of the same development period could be clustered together well, indicating a high similarity of the biological replicates (Figure 4).

### Target gene prediction and functional annotation

**Figure 5:**
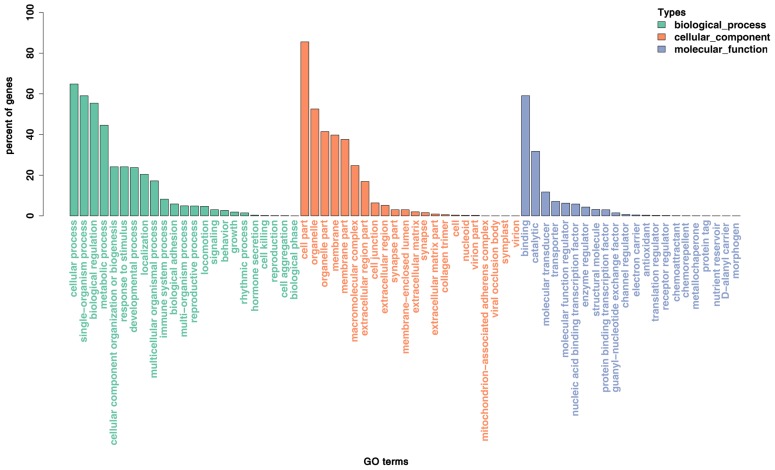
Enriched gene ontology (GO) terms of the targeted genes by the differentially expressed miRNAs between P70 and D70. P70, 70-day-old fetus; D70, 70-day-old piglets. X-axis, the GO terms names; Y-axis, the percent of genes enriched in this GO term. The histograms from left to right represent biological process, cellular-component and molecular-function in turn.

**Figure 6:**
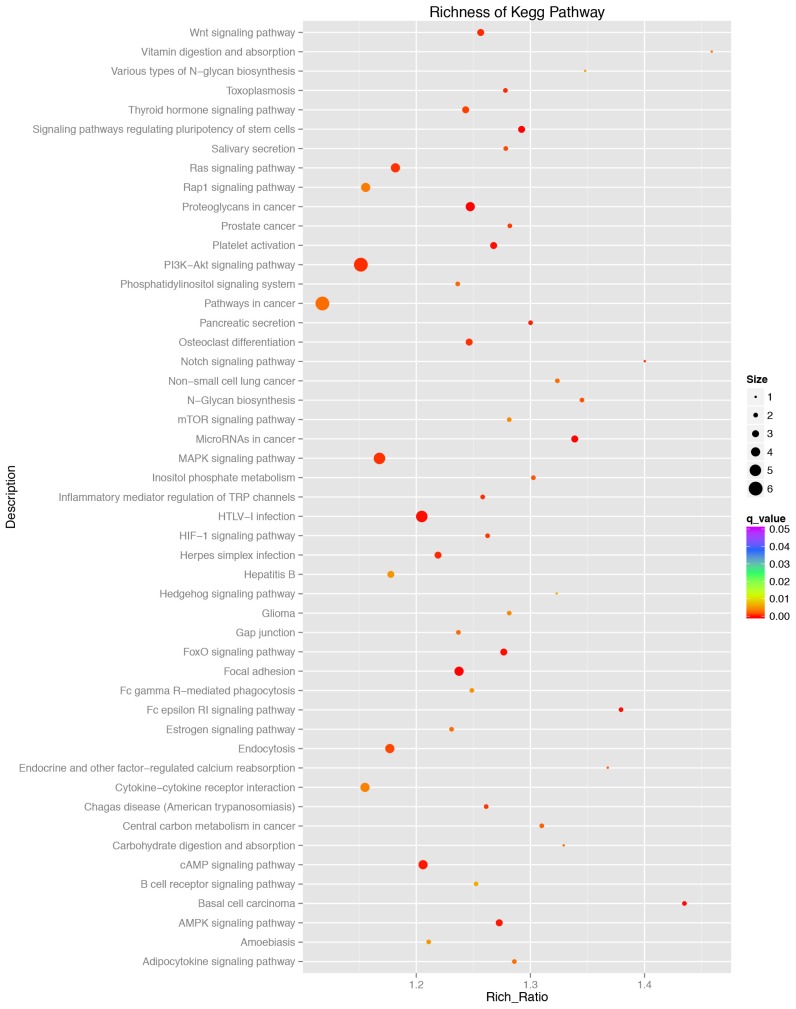
Pathway enrichment analysis of the targeted genes by the differentially expressed miRNAs between P70 and D70. X-axis, the richness ratio. Y-axis, the pathway name. The size of the circular, the number of genes enriched in this pathway. Only the significantly differential Kyoto encyclopedia of genes and genomes terms (q<0.05) were shown here. Red, high degree of enrichment. Purple, low degree of enrichment.

To better understand the function of miRNAs in the porcine liver lipid metabolism, it is essential to identify their target genes. We used TargetScan, miRanda, and TarBase to predict the target genes of the above 116 DE miRNAs. Then, all the target genes were subjected to GO and KEGG pathway enrichment analysis. The GO data showed that the DE miRNAs mainly participated in the cell growth, differentiation and development, regulation of intracellular signaling cascade, transcription regulation and metabolic process (Figure 5). Results of KEGG pathway enrichment analysis showed that the target genes were engaged in adipocytokine signaling pathway, mitogen-activated protein kinase (*MAPK*), AMPK, cyclic adenosine monophosphate (*cAMP*), phosphatidylinositol 3 kinase/protein kinase B (*PI3K-AKT*), Notch signaling pathway and the other well-known signaling pathways (Figure 6).

To identify the candidate miRNAs which may play vital roles in lipid metabolism, we integrated the miRNAs and mRNAs sequencing data (details of mRNAs sequencing data were shown in another unpublished paper). We found that several DE miRNAs (miR-181b, miR-145-5p, miR-199a-5p, and miR-98) directly targeted the functional genes which play important roles in lipid metabolism, such as acyl-CoA synthetase long chain family member 4 (*ACSL4*), ATP-binding casette A4 (*ABCA4*), and stearyl-CoA desaturase (*SCD*) (Table 3).

### Validation of the sequencing data, the lipid metabolism-associated DE miRNAs and their targeted genes by qRT-PCR

**Figure 7:**
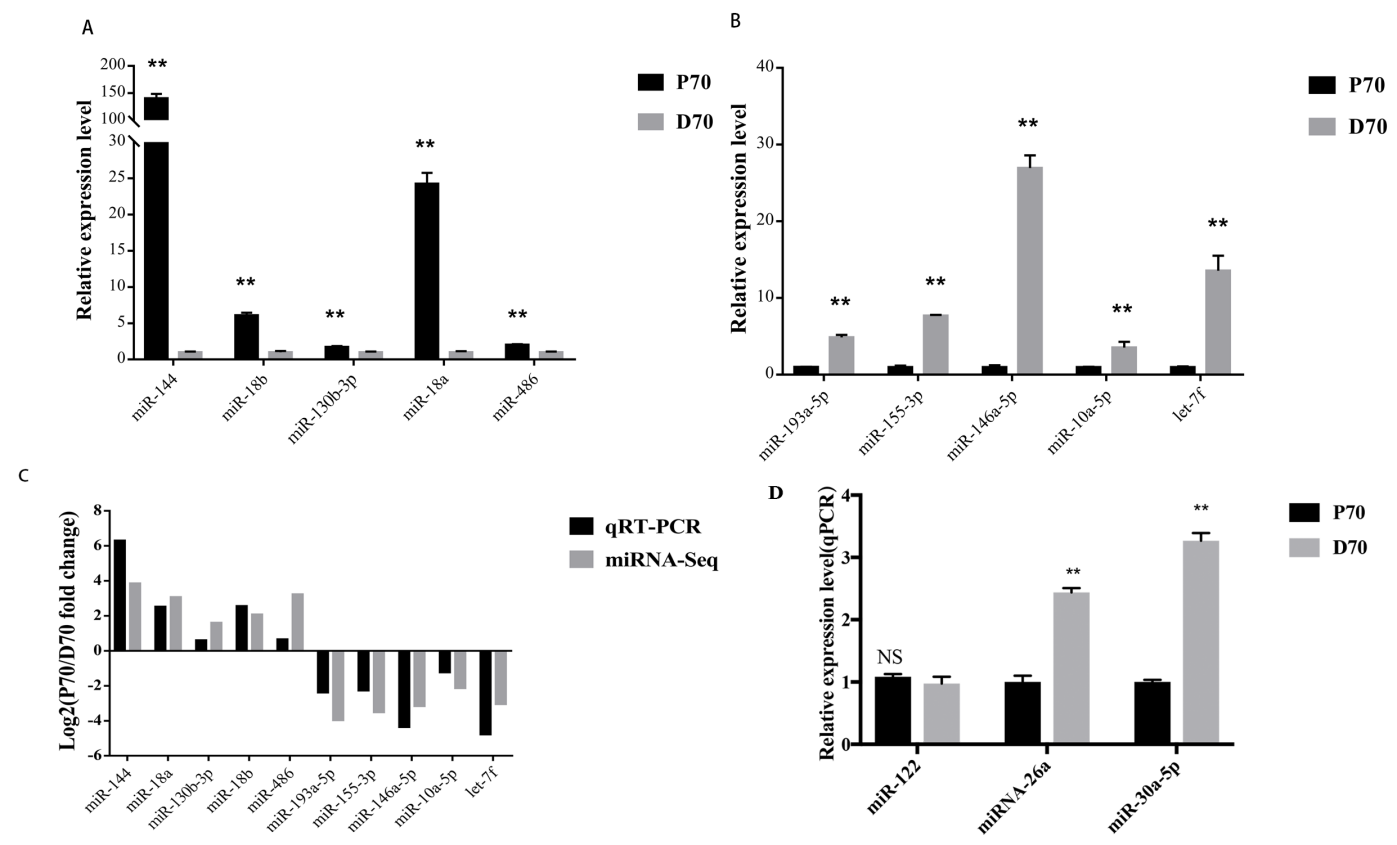
qRT-PCR validation of several DE miRNAs. * p<0.05; ** p<0.01. Data are shown as mean±standard error (SE). (A) 5 up-regulated miRNAs by qRT-PCR. X axis, relative expression level. Y axis, the up-regulated DE miRNAs. (B) 5 down-regulated miRNAs by qRT-PCR. X axis, relative expression level. Y axis, the down-regulated DE miRNAs. (C) comparison between the qRT-PCR results and the sequencing data. The X axis, the DE miRNAs. Y axis, the fold change between P70 and D70. (D) validation of the top abundantly expressed miRNAs by qRT-PCR. DE, differentially expressed; qRT-PCR, quantitative reverse-transcription polymerase chain reaction; X axis, relative expression level. Y axis, the top abundantly expressed miRNAs.

Ten DE microRNAs were randomly selected to validate the sequencing data by qRT-PCR. Among them, 5 miRNAs (miR-144, miR-18b, miR-130b-3p, miR-18a, miR-486) were up-regulated, and the others (miR-193a-5p, miR-155-3p, miR-146a-5p, miR-10a-5p, let-7f) were down-regulated. As shown in Figure 7A/B, the relative expression level of the selected miRNAs by qTR-PCR were significantly differentially expressed and consistent with the sequencing results (Figure 7C). The top abundantly expressed miRNAs (miR-122, miRNA-26a, and miR-30a-5p) were also validated by qRT-PCR (Figure 7D).

**Figure 8:**
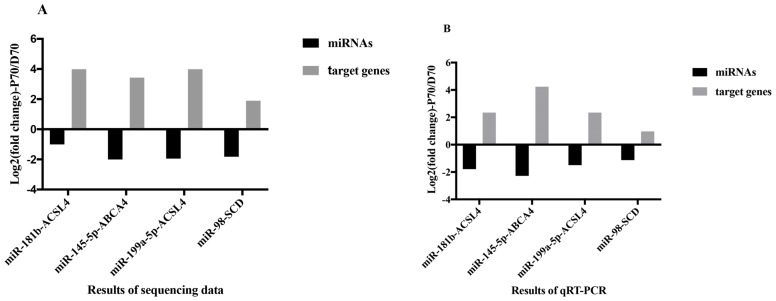
Sequencing data and qRT-PCR analysis of the potentially lipid-metabolism-related DE mRNAs and their target genes in P70 and D70 groups (n = 4 replicates per group). (A) Results of sequencing data. Black cylinders represent the diverse DE miRNAs respectively. Grey cylinders represent their target genes. (B) Results of the qRT-PCR. Black cylinders represent the diverse DE miRNAs respectively. Grey cylinders represent their target genes. qRT-PCR, quantitative reverse-transcription polymerase chain reaction; DE, differentially expressed.

In order to verify the relationship between the DE miRNAs and their target genes, qRT-PCR was employed to examine the expression levels of the four miRNAs and their corresponding target genes. As shown in Figure 8, consistent with expected inhibitory effects of miRNAs on their target genes, both qRT-PCR and sequencing (details of mRNA sequencing data were shown in another unpublished paper) results indicated that the expression levels of ACSL4, ABCA4, and SCD were significantly higher in P70, while the expression levels of miR-181b, miR-145-5p, miR-199a-5p, and miR-98 were significantly lower in P70.

## DISCUSSION

As the most important metabolic organ, the liver plays a critical role in fat deposition and lipid metabolism. Although the mechanisms of lipid metabolism in a variety of animal livers have been studied extensively, the potential molecular roles what the miRNAs may play in the lipid metabolism remain to be fully understood. It has been reported that the liver has different metabolic functions during the prenatal and postnatal stages. Although the fetal porcine liver between 40 and 80-day-old has certain metabolic functions, it mainly exercises intensive hematopoiesis [13,14]. In contrast, the liver of piglets mainly plays metabolic functions. Therefore, it is suitable to compare the miRNAome of the livers at P70 and D70, to obtain the candidate miRNAs that may be involved in porcine liver lipid metabolism.

In current study, we used high–throughput RNA sequencing technology to investigate the complexity of the porcine liver miRNA transcriptome in Large White at two devepomental periods, P70 and D70 respectively. In total, we constructed eight libraries, four from each physiological stage respectively. More than 179.6 million clear reads were obtained. Size distribution analyses showed that 22-nt RNAs were the most abundant, followed by 21- and 23-nt. The characteristics of length distribution is consistent with the typical size of microRNAs produced by Dicer processing and agrees with previous deep sequecing results [15]. All the above results indicated that the depth and quality of our sequencing data were reliable. Totally, we identified 346 known and 187 potential novel miRNAs. Most of the novel miRNAs were expressed at low levels, and had no expression differences between P70 and D70. Among the known miRNAs, the top three most abundantly expressed miR-122 (only in P70), miR-26a and miR-30 were reported to play significant roles in fatty acid biogenesis and metabolism. miR-122 was reported to be a liver-specific miRNA, and regulate a great number of genes associated with lipid metabolism in diverse species. A previous study in mice showed that circulating cholesterol was found to be lowered by 25% to 35% when miR-122 was inhibited [16]. In another study, it showed that the plasma cholesterol levels of the non-human primates were markedly reduced when miR-122 was antagonized [17]. MiR-122 exercised its function mainly through indirectly regulating the critical lipid metabolism functional genes, such as fatty acid synthase (*FASN*), microsomal triglyceride transfer protein (*MTP*) and *SCD*. *FASN* was found to play critical multi-functional roles in fatty acid synthesis and deposition [18]. *SCD* is a rate-limiting enzyme in catalyzing the synthesis of monounsaturated fatty acid [19]. In our current study, miR-122 showed no significant expression difference between P70 and D70. However, the miR-122 expression was high in both P70 and D70. miR-26a was also reported to be vital in regulating lipid metabolism by regulating the expression of ATP-binding casette A1 (*ABCA1*) and *ARL7*. *ABCA1* is a crucial factor for high-density lipoprotein synthesis and cholesterol efflux. It was found that the protein expression levels of ABCA1 and ARL7 were significantly down-regulated when miR-26 was over-expressed [20]. The opposite results were found while miR-26 was inhibited. Previous study has showed that miR-30c could lower plasma cholesterol by directly regulating *MTP* expression, which could further affect apolipoprotein B (*ApoB*) synthesis or secretion [21]. *ApoB* is primarily synthesized in hepatocytes and found to be an important regulator in lipoprotein metabolism [22]. Taken together, all the above findings indicate that miR-122, miR-26a, and miR-30 are critical regulators in White Large liver fundamental biological processes.

Among the known miRNAs, 116 were determined to be DE miRNAs. Hierarchical clustering of the DE miRNAs showed the miRNAs of the same developmental period could be clusterd into one group, which indicated a higher sample uniformity. GO functional enrichment analyses indicated that the DE miRNAs were mainly involved in the cell growth, differentiation and development, regulation of intracellular signaling cascade, transcription regulation and metabolic processes. Further study of KEGG pathway analyses showed that these DE miRNAs might exercise their functions through MAPK, PI3K-AKT, AMPK, cAMP, Wnt, Notch signaling pathway, adipocytokine signaling pathway and other well-known signaling pahways. Previous studies showed that the above mentioned multiple signaling pathways participated in hepatic lipid metabolism, including PI3K-AKT [23] and AMPK [24].

In this present study, ten DE miRNAs were randomly se lected to validate the sequencing data by qRT-PCR. Results of the qRT-PCR were confirmed to be consistent with the sequencing data. To further identify candidate miRNAs involved in porcine hepatic lipid metabolism, we integrated the miRNA and mRNA sequencing data (details of mRNAs sequencing data were shown in another unpublished paper). Based on the matching and energy stability assessment, we found 4 miRNA-mRNA pairs, including miR-181b-*ACSL4*, miR-145-5p-*ABCA4*, miR-199a-5p-*ACSL4*, and miR-98-*SCD*, which might participate in porcine lipid metabolism. Compared with the DE miRNAs, both qRT-PCR and sequencing results showed that the target genes observed in current study had the opposite expression trend between P70 and D70 groups (Figure 8). The result was consistent with the expected inhibitory effects of miRNAs on their target genes. Many previous studies showed that all the above mentioned miRNAs were related to adipogenesis and lipid metabolism in diverse species. Wang et al [25] found that miR-181b could mediate TG production in human livers by directly targeting NAD-dependent deacetylase sirtuin 1. In human hepatic and pancreatic islet cells, miR-145 was found to play an important role in regulating *ABCA1* expression [26]. miR-199a could impair mitochondrial fatty acid oxidation by targeting peroxisome proliferative activated receptor gamma (*PPARG*) [27]. *PPARG* is a vital transcription factor in regulating lipid metabolism. Chen et al [28] found that oleanolic acid could reduce the plasma TG and low-density lipoprotein by mediating miR-98 expression, which could down regulate peroxisome proliferator-activated receptor γ coactivator-1β, a vital regulator in maintaining hepatic homeostasis. In current study, it was found that miR-181b, miR-145-5p, miR-199a-5p, and miR-98 targeted *ACSL4*, *ABCA4*, *ACSL4*, and *SCD* respectively. *ACSL4* was found to play important roles in intracellular lipid storage and cholesterol transport [29]. *ABCA4* is one member of the ABC-transporter superfamily, and it has major effects on cellular cholesterol and lipoprotein metabolism [30]. SCD is a rate-limiting enzyme in catalyzing the synthesis of monounsaturated fatty acid [19]. Expression of *SCD* was found to be downregulated in D70, this may result in excess saturated fatty acid accumulation in the liver. All the above indicated that the 4 DE miRNAs might regulated lipid metabolism by attenuating *ACSL4*, *ABCA4*, and *SCD* respectively.

## CONCLUSION

In summary, our study has delineated the different porcine miRNA expression patterns of Large white livers at two different developmental stages (P70 and D70). An integrated analysis of miRNAs and the mRNA sequencing data revealed that 4 miRNA-mRNA pairs might be important regulators in porcine liver lipid metabolic process. It provides a valuable database for the further function and mechanism elucidation of miRNAs in porcine liver development and lipid metabolism.

## Figures and Tables

**Table 1:** miRNA primers used in this study

miRNA	miRNA specific forward primers sequence (5′- 3′)
miR-144	UACAGUAUAGAUGAUGUAC
miR-18a	UAAGGUGCAUCUAGUGCAGAUA
miR-193a-5p	UGGGUCUUUGCGGGCGAGAUGA
miR-155-3p	UCCUACAUGUUAGCAUUAACA
miR-146a-5p	UGAGAACUGAAUUCCAUGGGUU
miR-10a-5p	UACCCUGUAGAUCCGAAUUUGU
let-7f	UGAGGUAGUAGAUUGUAUAGUU
miR-130b-3p	CAGUGCAAUGAUGAUGAAAGGGCAU
miR-18b	UAAGGUGCAUCUAGUGCAGUUAG
miR-486	UCCUGUACUGAGCUGCCCCGAC
miR-181b	AACAUUCAUUGCUGUCGGUGGGUU
miR-145-5p	GUCCAGUUUUCCCAGGAAUCCCUU
miR-199a-5p	CCCAGUGUUCAGACUACCUGUUC
miR-98	UGAGGUAGUAAGUUGUAUUGUU
miR-122	UGGAGUGUGACAAUGGUGUUUGU
miR-26a	UUCAAGUAAUCCAGGAUAGGCU
miR-30a-5p	UGUAAACAUCCUCGACUGGAAG
U6	F: GCTTCGGCAGCACATATACT
	R: TTCACGAATTTGCGTGTCAT
ACSL4	F: TCTCCGCAGACACACCGATT
	R: GGTTTGGCTTGTCGTAGGCT
ABCA4	F: CGGGGTATACAGCGAACTCC
	R: CCACTCGAAATGAGGGCAGT
SCD	F: CCGTCAAAGAGAAGGGTGGT
	R: CAGCAATACCAGGGCACGAT
GAPDH	F: GGACTCATGACCACGGTCCAT
	R: TCAGATCCACAACCGACACGT

U6, small nuclear RNA U6; F, represents forward primers; R, represents reverse primers; ACSL4, Acyl-CoA synthetase long chain family member 4; ABCA4, ATP-binding casette A4; SCD, stearyl-CoA desaturase; GAPDH, glyceraldehyde-3-phosphate dehydrogenase.

**Table 2:** Summary of the high-throughput sequencing data

Sample	Raw reads	Clean reads	Percent (%)	Annotated reads	Perfect matches	Reads perfect matches in miRBase21.0 (Sus scrofa)
P70F1	29342610	23929565	81.55	22976029	16113027	10170227
P70F2	28418877	21820959	76.78	19784843	13814383	3773306
P70M1	28230328	23465932	83.12	19274238	14187701	5564433
P70M2	28084072	21408750	76.23	19820551	14683478	10714628
D70F1	26393049	23124241	87.61	22267257	15832481	12371347
D70F2	26046902	22462083	86.24	18828734	13360579	6310115
D70M1	25946611	21091073	81.29	19668363	13930426	7722316
D70M2	28966354	22286766	76.94	21148696	15379421	8014806

P70 represents liver samples from 70-day-old White Large fetus. D70 represents liver samples from 70-day-old White Large piglets. Percent represents clean reads/raw reads. Perfect matches represent the clean reads that completely matched the reference genome.

**Table 3:** miRNA-mRNA pairs potentially engaged in lipid metabolism

miRNA	Predicted target genes	mfe (kcal/mol)	Bingding sequences
miR-181b	*ACSL4*	−24.3	miRNA: 5′ AA***CAUUC***AUUGCUGUCGGUGGGUU3,
			target gene:3′ A***GUAAG***AUACAAACGGUCAUCUG5,
miR-145-5p	*ABCA4*	−26.7	miRNA: 5′ G***UCCAGUU***UUCCCAGGAAUCCCUU3,
			target gene: 3′ CC***GGUGUCGG***UGGUCCUCUACUUGGGU 5,
miR-199a-5p	*ACSL4*	−24.5	miRNA: 5′ C***CCAGU***G***U***UCAGACUACCUGUUC3,
			target gene: 3′ G***GGUUAA***AGAUCUAAAGGUGGAUG5,
miR-98	*SCD*	−22.2	miRNA: 5′ U***G***A***GGUAG***UAAGUUGUAUUGUU3,
			target gene: 3′ GA***CCCGUC***AGUCGGCUCACAGG 5,

*ACSL4*, Acyl-CoA synthetase long chain family member 4; *ABCA4*, ATP-binding casette A4; *SCD*, stearyl-CoA desaturase.

The sloping and bold letters indicate the miRNAs 2–8 seed sequence which are complementary to the target genes.
