# Supplementary material for: Hepatic microRNAome reveals potential microRNA-mRNA pairs association with lipid metabolism in pigs
Source: Asian-Australas J Anim Sci. 2018 Sep 13;32(9):1458–68. doi: 10.5713/ajas.18.0438 (PMC6722318; doi:10.5713/ajas.18.0438)
Supplement: Supplementary file 1 [file ajas-18-0438-suppl.pdf]

Table S1. Summary of the acquired miRNAs in porcine liver.

known miRNAs(numbers)	pre-miRNAs(numbers)	up-regulated(numbers)	down-regulated(numbers)	novel miRNAs(numbers)	pre-miRNAs*(numbers)	up-regulated*(numbers)	down-regulated*(numbers)
346	279	61	55	187	183	0	0

Pre-miRNAs and pre-miRNAs* indicate precursor-miRNAs of known and novel miRNAs respectively. Up-regulated, down-regulated, up-regulated* and down-regulated* indicate differential expressed miRNAs of known and novel miRNAs respectively.

Excel S1 Details of known miRNAs-count

miRNA_Name	D70F1	D70F2	D70M1	D70M2	P70F2	P70F1	P70M1	P70M2
ssc-let-7c	3451	1465.58	1847	1603.05	1307	455.83	537.7	1185.3
ssc-let-7d-3p	28.17	18.79	19.81	22.17	20.67	12.38	11.7	17.43
ssc-let-7d-5p	1355	516.29	610.8	636.3	228.8	74.92	107.1	239.11
ssc-let-7e	669.6	238.61	353.9	258.85	478.9	238.09	255.9	413.87
ssc-let-7f	32948	15078.4	19818	14198.7	6398	2429	2628	6719
ssc-let-7g	7501	4301.83	4719	4479.17	1533	712.08	1088	1739.1
ssc-miR-100	1332	635	733.5	994.38	1723	1064.7	1086	2479
ssc-miR-103	1599	1002.95	1272	982.09	4796	1613.8	2315	4990.6
ssc-miR-105-1	0.06	0	0	0	0.25	0.14	0	0.07
ssc-miR-105-2	0	0	0	0.07	0.06	0	0.07	0
ssc-miR-106a	0.82	0.6	0.22	0.33	3.97	2.1	1.9	4.29
ssc-miR-107	700	331.57	415.1	492.48	2766	881.33	828.5	3169.8
ssc-miR-10a-3p	45.54	15.12	21.18	29.19	24.89	9.77	8.74	22.75
ssc-miR-10a-5p	9176	4729.21	9881	4580.86	3633	1819.8	2693	3203.2
ssc-miR-10b	461.9	177.16	1062	244.42	1093	485.65	567.7	253.07
ssc-miR-122	18498	8067.02	4354	8308.18	47433	10228	14855	10631
ssc-miR-1224	0	0	0.07	0	0	0	0	0
ssc-miR-1249	1.77	1.57	0.86	0.98	0.62	1.23	0.21	0.14
ssc-miR-124a	0	0	0.07	0	0.37	0.65	0.56	0.14
ssc-miR-125a	3701	1949.62	3509	2211.72	4955	3449.3	4642	5385.4
ssc-miR-125b	8257	4012.7	5755	5372.63	5007	2850.1	3046	5914.5
ssc-miR-126-3p	9503	4836.39	5057	6081.05	11338	5788.3	5734	10050
ssc-miR-126-5p	0	0.07	0	0.07	0.06	0	0	0
ssc-miR-127	61.83	41.24	107.6	79	986.8	720.34	545.6	1654.2
ssc-miR-1271	1.83	0.9	0.86	2.02	4.84	2.75	2.89	6.95
ssc-miR-1296-3p	0	0	0	0	0	0	0	0
ssc-miR-1296-5p	19.96	9.51	9.91	16.78	97.13	45.46	63.65	117.62
ssc-miR-129a-3p	0	0	0	0	0	0	0	0
ssc-miR-129a-5p	0	0	0	0	0	0	0	0
ssc-miR-129b	0	0	0	0	0	0	0	0
ssc-miR-1306-3p	2.08	0.97	1.08	1.43	8.38	3.69	3.17	12.67
ssc-miR-1306-5p	6.57	3.22	3.23	4.88	28.18	13.68	13.81	24.86
ssc-miR-1307	80.97	43.56	49.32	41.16	184.5	98.23	88.81	196.34
ssc-miR-130a	0	0	0	0	0	0	0	0
ssc-miR-130b	168.3	82.71	112.6	102.28	833.8	294.69	464.5	1064.9
ssc-miR-132	12.13	2.92	2.3	2.93	5.71	2.97	2.82	5.04
ssc-miR-133b	0.76	0.3	0.36	0.2	0.62	0.72	0.7	1.36
ssc-miR-1343	23.24	13.85	18.31	13.2	57.1	39.67	30.03	58.84
ssc-miR-135	0	0	0	0	0	0	0	0
ssc-miR-136	0	0	0	0	0	0	0	0
ssc-miR-137	0.88	0.75	1	0.91	9.12	11	10.85	5.11
ssc-miR-139-3p	2.59	1.12	1.58	1.11	2.3	1.45	2.04	2.59
ssc-miR-139-5p	188.7	120.28	137.4	137.13	179.1	61.75	114.8	204.72

ssc-miR-140-3p	2217	1191.79	1150	1211.62	1730	1075	1019	2031.9
ssc-miR-140-5p	0	0	0	0	0	0	0.07	0
ssc-miR-142-3p	765.1	600.12	576.1	444.43	994.1	612.77	997.4	980.56
ssc-miR-142-5p	9581	6011.79	4506	8660.47	11170	6400.6	7417	15082
ssc-miR-143-3p	19326	9172.96	10891	17186.1	15176	11124	7107	19519
ssc-miR-143-5p	10.23	4.19	5.46	7.74	8.25	5.5	4.16	9.13
ssc-miR-144	386.5	64.74	147.8	271.14	6028	4130.1	2477	9234.7
ssc-miR-145-3p	136.7	63.84	99.35	117.3	50.15	21.57	29.32	66.06
ssc-miR-145-5p	1.07	0.6	0.72	0.33	0.37	0.07	0.42	0.34
ssc-miR-1468	0	0	0	0	0	0	0	0
ssc-miR-146a-3p	0	0	0	0.07	0	0	0	0
ssc-miR-146a-5p	347.3	299.24	217.4	239.02	65.1	38.37	56.25	55.71
ssc-miR-146b	64.42	94.08	35.61	28.48	46.24	31.34	52.37	30.37
ssc-miR-148b-3p	463	289.66	323.5	256.38	1347	407.04	811	1471.9
ssc-miR-148b-5p	85.84	58.53	99.85	52.6	215	63.2	165.9	229.31
ssc-miR-149	9.47	5.69	7.39	12.35	13.9	7.46	8.25	11.17
ssc-miR-150	303.4	347.14	281.8	372.45	169	194.07	212.3	102.77
ssc-miR-151-3p	1840	989.85	1458	1275.21	1624	1166.3	901	2159.2
ssc-miR-152	659.1	323.64	132.3	433.18	3160	732.28	800.7	1329.9
ssc-miR-155-3p	4.11	0.9	0.86	0.59	0.31	0.22	0	0.41
ssc-miR-15a	1467	730.81	627	980.6	1678	508.82	805.1	2068.5
ssc-miR-15b	138.2	75.3	89.59	88.43	279.7	85.85	220.8	400.31
ssc-miR-16	21649	11090.2	10390	11875.4	30478	8307.9	12874	35533
ssc-miR-17-3p	41.24	22.53	24.84	23.02	135.4	49.01	90.01	161.27
ssc-miR-17-5p	358.3	247	275.7	182.26	1831	617.76	1640	1936.4
ssc-miR-181b	1437	824.52	1101	730.26	1222	445.55	732	1400
ssc-miR-181c	444.4	203.06	290.7	312.3	748.5	334.14	489.7	750.91
ssc-miR-181d-3p	0.06	0	0	0	0.06	0	0.07	0.27
ssc-miR-181d-5p	37.9	18.26	26.85	23.99	78.69	32.57	52.16	74.16
ssc-miR-182	390.2	168.78	506.3	235.12	1223	924.54	1080	1150.5
ssc-miR-183	5.5	3.22	3.95	4.88	86.7	57.91	65.62	75.19
ssc-miR-1839-3p	0	0	0	0	0	0	0	0
ssc-miR-1839-5p	3.66	2.4	3.37	2.6	10.67	7.75	10.78	9.88
ssc-miR-184	5.94	6.21	6.89	2.8	1102	1001.6	920.4	1595.2
ssc-miR-185	9.66	5.54	6.82	6.18	14.89	11.08	11.35	15.87
ssc-miR-186	1.64	1.65	1.08	0.98	1.37	0.51	2.47	1.23
ssc-miR-187	0.44	0.3	0.07	0.39	0.99	0.43	0.21	0.54
ssc-miR-18a	23.62	16.92	15.72	16.13	321.7	137.75	296.9	341.47
ssc-miR-18b	1.96	1.65	1.36	3.32	22.47	10.13	6.7	30.1
ssc-miR-190a	11.75	9.8	8.97	8.19	29.48	15.49	23.4	23.09
ssc-miR-190b	0.44	0.22	0.07	0	0.43	0.51	0.85	0.95
ssc-miR-191	10.42	10.48	16.01	5.53	3.97	1.52	5.78	3.27
ssc-miR-192	1E+05	83225.7	98946	114848	52934	27211	33046	89249
ssc-miR-193a-3p	87.98	34.88	56.78	44.28	17.32	6.73	5.85	10.42
ssc-miR-193a-5p	1.58	1.42	1.87	1.76	0.43	0.07	0.14	0.2

ssc-miR-194a	21242	12693.1	13949	15269.8	10099	3228.5	5206	13426
ssc-miR-194b-3p	0.06	0.07	0	0	0	0	0	0
ssc-miR-194b-5p	2.46	3.67	1.79	3.19	1.3	0.36	0.92	2.11
ssc-miR-195	362.5	186.29	119	187.72	78.57	28.96	43.91	99.64
ssc-miR-196b-3p	0	0	0	0	0	0	0	0.07
ssc-miR-199a-3p	20356	8716.91	13597	13817.1	9024	2288.6	3536	9876.9
ssc-miR-199a-5p	4549	2502.66	4769	3144.07	2109	1333.5	1333	2110.7
ssc-miR-199b-3p	20356	8716.91	13597	13817.2	9024	2288.6	3536	9877
ssc-miR-199b-5p	1032	522.96	941.2	563.42	633.8	185.1	315.9	437.57
ssc-miR-19a	0.32	0.15	0.14	0	0.93	0.07	0.85	0.07
ssc-miR-202-3p	0	0	0	0	0.06	0	0	0
ssc-miR-202-5p	0.19	0	0.07	0.07	2.36	0.72	0.78	0.27
ssc-miR-204	928.7	518.17	488.1	679.41	2461	292.3	468.7	1781.7
ssc-miR-205	2.78	1.8	2.73	3.06	33.89	6.37	21.99	17.84
ssc-miR-206	0	0	0	0	0	0	0.07	0.2
ssc-miR-208b	0	0	0	0	0	0	0	0
ssc-miR-20a	489.6	327.46	371.9	246.69	1364	692.47	1676	1396.9
ssc-miR-20b	3.85	2.47	1.94	3.97	18.43	6.66	11.63	16.41
ssc-miR-21	21787	13846.6	20099	10204.7	5852	3054.1	5236	6162
ssc-miR-210	2.4	1.95	1.79	2.15	20.36	18.53	14.94	27.31
ssc-miR-212	1.71	0.75	0.29	0.46	1.99	1.45	1.2	1.63
ssc-miR-214	236.7	105.31	152.6	152.74	110.5	33.15	45.74	87.58
ssc-miR-215	71.62	54.26	81.48	132.32	15.89	14.48	5.07	48.76
ssc-miR-216	0.63	0.22	0.14	0.52	12.04	5.79	8.53	9.88
ssc-miR-217	0.57	0.07	0.36	0.26	14.77	5.36	10.43	10.15
ssc-miR-218	56.78	28.89	32.02	70.94	69.7	39.52	23.89	77.91
ssc-miR-218	56.78	28.89	32.02	70.94	69.7	39.52	23.89	77.91
ssc-miR-218-5p	56.78	28.89	32.02	70.94	69.7	39.52	23.89	77.91
ssc-miR-218-5p	56.78	28.89	32.02	70.94	69.7	39.52	23.89	77.91
ssc-miR-218b	56.59	28.67	31.8	70.87	69.63	39.45	23.82	77.71
ssc-miR-219a	2.53	1.05	1.79	1.3	3.41	1.23	1.97	4.22
ssc-miR-219b-3p	0.13	0.15	0.29	0	0.12	0	0.07	0.07
ssc-miR-221-3p	866.3	574.9	668.1	628.83	2399	1163.7	1748	3359
ssc-miR-221-5p	48	47.9	82.48	24.45	142.8	37.35	158.2	137.09
ssc-miR-222	742.2	678.11	844	297.41	6319	1676.2	3409	6333.6
ssc-miR-224	0.38	0	0.5	0.2	0.56	0.36	0.49	0.48
ssc-miR-2320-3p	3.98	2.62	1.79	2.34	10.3	3.98	2.89	10.9
ssc-miR-2320-5p	5.94	3.67	5.67	2.67	16.14	9.41	12.97	18.86
ssc-miR-23a	340.4	215.71	285.2	246.04	150.2	71.16	111.3	150.17
ssc-miR-23b	558.6	265.93	263.1	342.21	318.4	99.61	148	332.35
ssc-miR-24-1-5p	23.56	16.47	26.2	11.05	14.77	7.38	15.72	16.82
ssc-miR-24-2-5p	4.29	2.69	3.59	2.21	2.48	1.74	1.48	1.5
ssc-miR-24-3p	512	194.68	164	288.5	425.2	118.86	131	221
ssc-miR-2411	1.01	0.22	0.72	0.39	3.04	1.45	1.97	4.02
ssc-miR-2483	1.64	0.45	1.44	1.56	7.2	2.82	2.33	13.08

ssc-miR-26a	91013	58348.7	69175	61464.4	62546	21346	32474	74775
ssc-miR-27a	945.6	402.38	491.4	674.47	253.4	159.62	115.5	309.8
ssc-miR-27b-3p	8927	4012.03	5415	6348.42	5993	2172.2	2197	7945.2
ssc-miR-27b-5p	12.7	9.58	10.41	7.22	12.97	6.01	7.05	15.32
ssc-miR-28-5p	678.7	294.52	383.6	456.78	817.4	198.92	271.1	856.47
ssc-miR-296-3p	25.39	13.4	20.17	13.46	355.2	133.7	139.6	430.82
ssc-miR-296-5p	5.56	2.92	1.87	3.19	23.15	11.58	8.67	21.38
ssc-miR-29a	7186	4544.26	5062	5107.99	2781	1629.1	2146	4063.7
ssc-miR-29b	153.4	130.68	201.5	103.78	134.6	79.92	141.2	155.69
ssc-miR-29c	314.6	190.86	180.1	193.12	166.4	127.98	143.7	164.67
ssc-miR-30a-3p	368.1	243.18	242.6	252.87	462.1	239.75	434.5	596.04
ssc-miR-30a-5p	32895	24359.7	32288	20367.2	25826	14252	22967	28719
ssc-miR-30b-3p	7.83	5.46	5.46	6.24	15.58	6.23	8.18	14.91
ssc-miR-30b-5p	765.4	550.95	506.5	632.99	827.8	326.76	689.5	711.21
ssc-miR-30c-1-3p	23.94	16.02	20.03	20.29	75.9	32.86	37.99	75.87
ssc-miR-30c-3p	73.27	42.44	59.22	44.74	161.9	112.49	99.8	179.39
ssc-miR-30c-5p	880.8	877.73	982.3	781.5	1969	708.75	2198	1246.6
ssc-miR-30e-3p	251	145.35	160.7	164.51	313.5	127.48	242.7	339.84
ssc-miR-30e-5p	17727	13537.4	19829	9949.33	16129	7271.8	16131	17435
ssc-miR-31	3.6	2.32	1	2.86	5.28	4.56	2.4	11.78
ssc-miR-32	195	94.16	119.2	167.11	133.9	52.19	69.78	134.1
ssc-miR-320	15.73	6.59	8.76	8.52	30.04	14.62	12.97	31.06
ssc-miR-324	35.81	15.94	21.82	22.24	145.4	67.61	99.24	138.25
ssc-miR-326	3.09	1.57	1.87	2.54	4.47	4.56	1.97	6.27
ssc-miR-328	14.84	7.93	9.55	8.91	29.85	17.74	15.51	30.1
ssc-miR-331-3p	23.18	12.13	17.95	19.83	35.87	37.35	16.28	43.04
ssc-miR-331-5p	23.05	12.05	16.65	13.78	17.32	7.82	11.14	20.16
ssc-miR-335	0	0	0	0	0	0	0	0
ssc-miR-338	0	0	0	0	0	0	0	0
ssc-miR-339-3p	6	2.25	2.8	3.12	4.53	3.62	1.76	5.72
ssc-miR-345-3p	3.47	1.87	1.72	2.02	4.41	2.9	3.95	3.68
ssc-miR-345-5p	3.92	2.47	2.8	3.64	6.27	4.85	3.59	8.24
ssc-miR-34c	12.88	7.11	14.72	10.73	31.84	18.02	34.54	37.39
ssc-miR-361-3p	172	96.85	147.5	88.75	91.04	44.81	63.58	87.51
ssc-miR-361-5p	225.7	120.13	152.3	145.71	231.7	96.49	159.8	295.43
ssc-miR-362	9.6	6.44	11.84	5.79	56.91	32.07	80.21	76.28
ssc-miR-363	30.63	21.63	23.69	33.68	128.3	43.14	58.5	156.91
ssc-miR-365-3p	0	0	0	0	0	0	0	0
ssc-miR-365-5p	0.51	0.22	0.07	0.2	0.12	0	0.14	0
ssc-miR-371-5p	0.38	0	0	0.52	0.12	0.07	0	0
ssc-miR-374a-3p	493.4	290.86	347.7	330.77	418.1	177.42	254.9	506.83
ssc-miR-374a-5p	663.8	416.15	402.4	430.71	788.7	325.68	510	715.36
ssc-miR-374b-3p	20.72	12.57	14.07	13.91	46.48	12.16	26.57	59.18
ssc-miR-374b-5p	124.1	96.48	97.48	98.18	294.7	138.91	296.9	348.28
ssc-miR-376a-3p	0	0	0	0	0	0	0	0

ssc-miR-376a-5p	1.14	0.97	0.79	0.65	68.02	36.56	32.63	78.12
ssc-miR-376c	0	0	0	0	0.93	0.43	0.07	0.41
ssc-miR-378	1149	572.58	713.8	713.36	536.8	349.35	488.4	707.46
ssc-miR-378b-3p	67.08	28.37	38.62	59.04	26.69	25.77	13.53	45.02
ssc-miR-381	0.19	0.22	0.07	0	3.79	1.23	2.11	3.41
ssc-miR-383	0	0	0	0	0.06	0	0	0.14
ssc-miR-421-3p	49.52	30.46	38.05	30.63	321.5	105.33	203.6	417.41
ssc-miR-421-5p	0.95	0.15	0.72	0.46	3.48	1.66	2.68	5.31
ssc-miR-423-3p	1110	640.54	892.7	582.66	3415	958.13	1485	3425.9
ssc-miR-423-5p	754.5	364.13	842.3	289.35	522.1	207.1	211.5	323.97
ssc-miR-425-3p	186.2	95.06	98.56	144.02	212.9	94.76	101.1	163.65
ssc-miR-425-5p	656	466.75	654.1	588.25	1174	357.67	622.7	910.55
ssc-miR-429	31.71	11.6	10.19	24.77	141.4	63.12	60.9	154.87
ssc-miR-432-3p	0	0	0.07	0	0.5	0.14	0.14	0.54
ssc-miR-432-5p	1.45	0.9	2.58	1.5	42.2	16.21	21.57	45.7
ssc-miR-4334-3p	413.8	231.43	236	479.86	459.5	328.64	177.6	748.73
ssc-miR-4334-5p	0	0	0	0	0	0	0	0
ssc-miR-4337	0	0	0.14	0	0.31	0	0.14	0.14
ssc-miR-4338	0.19	0	0	0.2	0	0	0	0
ssc-miR-450a	0.13	0.07	0	0.07	0.25	0.36	0.21	0.75
ssc-miR-450b-3p	0	0	0	0	0	0	0	0
ssc-miR-450b-5p	0.63	0.22	0.5	0.52	4.34	1.95	1.62	6.06
ssc-miR-450c-3p	0	0	0	0	0	0	0	0
ssc-miR-450c-5p	0	0.07	0.22	0	0.68	0.65	0.49	1.02
ssc-miR-451	2216	566.22	1005	745.22	8772	5537.6	12107	11316
ssc-miR-452	1.14	0.22	0.65	0.07	0.99	0.36	0.49	0.14
ssc-miR-486	198	31.06	104.5	104.17	2147	1094.7	1216	2595.6
ssc-miR-489	0.06	0	0	0	0	0	0	0.07
ssc-miR-490	6.32	3.82	6.75	3.32	14.09	14.55	11	16.28
ssc-miR-490	6.32	3.82	6.75	3.32	14.09	14.55	11	16.28
ssc-miR-490-3p	6.32	3.82	6.75	3.32	14.09	14.55	11	16.28
ssc-miR-490-3p	6.32	3.82	6.75	3.32	14.09	14.55	11	16.28
ssc-miR-490-5p	0.19	0	0.14	0.2	0.81	1.38	0.42	1.7
ssc-miR-490-5p	0.19	0	0.14	0.2	0.81	1.38	0.42	1.7
ssc-miR-491	1.39	0.6	0.22	0.65	1.43	0.8	1.06	1.63
ssc-miR-493-3p	0.19	0.07	0.22	0.26	4.9	2.46	2.11	6.95
ssc-miR-493-5p	0.63	0.15	0.65	0.85	15.95	3.55	6.2	13.01
ssc-miR-497	907.6	434.26	352.3	522.13	389.5	142.03	219.6	462.77
ssc-miR-499-3p	2.27	1.72	2.01	2.41	3.6	1.59	1.97	5.31
ssc-miR-499-5p	48.26	28.29	30.29	38.69	67.09	28.96	32.99	75.94
ssc-miR-500	53.43	41.39	66.62	31.93	643.2	485.36	769.9	864.99
ssc-miR-504	1.77	1.05	0.57	0.98	4.41	1.45	0.92	1.02
ssc-miR-505	226.3	148.95	199.6	143.11	206.5	59.43	113.4	226.44
ssc-miR-532-3p	7.58	3.89	4.09	5.07	80.18	55.74	62.59	86.83
ssc-miR-532-5p	164.9	94.76	96.19	114.5	1124	962.84	930.7	1633.2

ssc-miR-542-3p	103.1	44.76	65.18	79.39	678	308.52	403	743.15
ssc-miR-542-5p	29.31	10.63	21.03	18.21	204.9	68.62	88.74	192.67
ssc-miR-545-3p	66.51	46.85	48.02	43.37	208.7	99.03	128.1	226.58
ssc-miR-551a	1.07	0.6	0.22	0.91	7.26	5.36	5.15	10.35
ssc-miR-574	166.3	82.56	91.45	153.97	236.8	92.73	117.3	204.04
ssc-miR-582	16.67	10.18	17.16	13.98	89.18	28.59	56.74	41.88
ssc-miR-615	0	0	0	0	0	0.07	0	0.14
ssc-miR-628	34.36	16.99	25.99	19.44	104.8	26.57	46.52	102.16
ssc-miR-652	0.13	0.07	0	0.13	0.19	0.07	0.42	0.27
ssc-miR-664-3p	7.33	6.96	8.18	5.59	7.51	3.91	5.99	5.04
ssc-miR-664-5p	56.53	34.21	51.25	30.24	75.59	35.76	45.74	78.18
ssc-miR-671-3p	8.02	3.97	2.94	4.16	10.61	4.2	5.15	15.12
ssc-miR-671-5p	7.64	3.97	7.18	3.32	12.35	4.99	11.84	10.01
ssc-miR-676-3p	27.22	8.46	14	16.32	42.14	17.23	12.33	46.72
ssc-miR-676-3p	27.22	8.46	14	16.32	42.14	17.23	12.33	46.72
ssc-miR-676-5p	0	0.07	0	0.07	0.25	0.14	0	0.48
ssc-miR-676-5p	0	0.07	0	0.07	0.25	0.14	0	0.48
ssc-miR-6782-3p	1.77	0.52	0.57	1.69	2.11	1.81	2.33	1.23
ssc-miR-7	0.19	0.15	0.07	0.07	0.56	0.29	0.21	0.2
ssc-miR-708-3p	1.39	0.37	0.86	0.72	4.59	1.66	1.06	2.52
ssc-miR-708-5p	8.72	1.87	3.16	3.25	27.56	5.72	10.71	12.6
ssc-miR-7134-3p	871.6	397.29	404.5	543.19	795	323.14	225.4	787.55
ssc-miR-7134-5p	20.46	13.1	10.05	21.26	36.37	28.09	26.22	51.9
ssc-miR-7135-3p	0.38	0.37	0.07	0.33	0.06	0.14	0.07	0
ssc-miR-7135-5p	0.06	0.07	0.07	0.26	0.12	0	0.07	0.07
ssc-miR-7136-3p	0.06	0	0	0	0.06	0.07	0.07	0.07
ssc-miR-7137-3p	2.53	0.6	0.65	0.39	1.74	1.74	1.76	1.36
ssc-miR-7137-5p	3.85	1.57	1.94	3.25	7.08	5.21	4.72	7.49
ssc-miR-7138-3p	0.32	0.15	0.07	0	1.24	0.65	0.42	1.91
ssc-miR-7138-5p	1.14	1.35	1.08	1.63	4.59	4.49	6.91	4.49
ssc-miR-7139-3p	0.95	0.6	0.07	0.59	3.6	1.38	0.99	3.68
ssc-miR-7139-5p	2.72	1.35	3.59	4.42	28.18	7.31	11	30.58
ssc-miR-7140-5p	0	0	0	0	0	0	0	0.07
ssc-miR-7141-3p	0	0	0	0	0	0	0	0
ssc-miR-7141-5p	0	0	0	0	0	0	0	0
ssc-miR-7142-3p	74.53	43.64	65.25	53.9	94.71	107.42	88.39	161.27
ssc-miR-7142-5p	0	0	0.07	0	0.12	0.07	0.07	0
ssc-miR-7144-3p	0	0	0	0	0	0	0	0
ssc-miR-7144-5p	2.78	1.05	1.29	0.98	9.12	3.62	6.34	7.36
ssc-miR-744	30.51	18.41	14.79	24.19	255.8	97.58	95.01	249.12
ssc-miR-769-5p	148.1	85.55	90.23	110.08	330.8	225.49	233.2	400.86
ssc-miR-7857-3p	0.06	0	0.07	0	1.24	0.36	0.35	1.02
ssc-miR-874	172.4	86.45	129.5	78.55	508.3	322.2	308.4	626.15
ssc-miR-9	15.6	9.95	11.84	10.79	21.41	23.24	24.18	10.9
ssc-miR-9-1	15.6	9.95	11.84	10.79	21.41	23.24	24.18	10.9

ssc-miR-9-2	15.6	9.95	11.84	10.79	21.41	23.24	24.18	10.9
ssc-miR-92b-3p	64.49	57.63	43.86	53.97	115.6	44.37	49.13	117.62
ssc-miR-92b-5p	0.25	0.22	0.43	0.13	0.43	0.07	0.35	0.41
ssc-miR-935	0.19	0.07	0.07	0.26	0.31	0.07	0.14	0.14
ssc-miR-95	57.1	30.46	24.19	27.76	17.44	3.33	7.12	11.44
ssc-miR-96-5p	0	0	0	0	0	0	0	0
ssc-miR-9785-5p	0.06	0.15	0.07	0.07	0.37	0.22	0.14	0.14
ssc-miR-9786-3p	0	0	0	0	0	0	0	0
ssc-miR-9788-3p	0.13	0	0	0.07	0.06	0.07	0.21	0.14
ssc-miR-9789-3p	0	0	0	0	0	0	0	0
ssc-miR-9790-3p	0	0	0	0	0	0	0	0
ssc-miR-9791-3p	0	0	0	0	0	0	0	0
ssc-miR-9792-5p	0	0	0	0	0	0	0	0
ssc-miR-9793-5p	0	0	0	0	0	0	0	0
ssc-miR-9794-3p	0	0	0	0	0	0	0	0
ssc-miR-9795-3p	0	0	0	0	0	0	0	0
ssc-miR-9796-3p	0	0	0	0	0	0	0	0
ssc-miR-9797-3p	0	0	0	0	0	0	0	0
ssc-miR-9798-3p	0	0	0	0	0	0	0	0
ssc-miR-9799-3p	0	0	0	0	0	0	0	0
ssc-miR-98	627.1	396.09	444.2	452.81	291.8	178.94	175.2	328.4
ssc-miR-9800-3p	0	0	0	0	0	0	0	0
ssc-miR-9801-5p	0	0	0	0	0	0	0	0
ssc-miR-9802-3p	0	0	0	0	0	0	0	0
ssc-miR-9803-5p	0	0	0	0	0	0	0.07	0
ssc-miR-9804-5p	0	0	0	0	0	0	0	0
ssc-miR-9805-3p	0	0	0	0	0	0	0	0
ssc-miR-9807-5p	0	0	0	0	0	0	0	0
ssc-miR-9808-3p	0	0	0	0	0	0	0	0
ssc-miR-9809-3p	0	0	0	0	0	0	0	0
ssc-miR-9810-3p	0.06	0.15	0.14	0.26	3.29	1.01	0.92	2.45
ssc-miR-9811-5p	0	0.07	0	0	0	0	0	0
ssc-miR-9812-3p	0	0	0	0	0.06	0	0	0
ssc-miR-9813-5p	0	0	0	0	0	0	0	0
ssc-miR-9814-3p	0	0	0	0	0	0	0	0
ssc-miR-9815-3p	0	0	0	0	0	0	0	0
ssc-miR-9816-3p	0	0	0	0	0	0	0	0
ssc-miR-9817-5p	0	0	0	0	0	0	0	0
ssc-miR-9818-3p	0	0	0	0	0	0.07	0	0
ssc-miR-9819-5p	0	0	0	0	0	0	0	0
ssc-miR-9820-5p	0	0.07	0	0	0.06	0	0	0.2
ssc-miR-9821-5p	0	0	0	0	0	0	0	0
ssc-miR-9822-3p	0	0	0	0	0	0	0	0
ssc-miR-9823-5p	0	0	0	0	0	0	0	0
ssc-miR-9824-5p	0	0	0	0	0.06	0	0	0

ssc-miR-9825-5p	0	0	0	0	0	0	0	0
ssc-miR-9826-5p	0	0	0	0	0	0	0	0
ssc-miR-9827-5p	0	0	0	0	0	0	0	0
ssc-miR-9829-5p	0	0	0	0	0	0	0	0
ssc-miR-9830-5p	0	0	0	0	0	0	0	0
ssc-miR-9831-3p	0	0	0	0	0	0	0	0
ssc-miR-9832-3p	0	0	0	0	0	0	0	0
ssc-miR-9833-5p	0	0	0	0	0	0.07	0	0
ssc-miR-9834-5p	0	0	0	0	0	0	0	0
ssc-miR-9835-3p	0	0	0	0	0	0	0	0
ssc-miR-9836-3p	0	0	0	0	0	0	0	0
ssc-miR-9837-5p	0	0	0	0	0	0	0	0
ssc-miR-9838-5p	0	0	0	0	0	0	0	0
ssc-miR-9841-3p	7.2	4.34	3.52	7.35	25.57	13.46	10.92	30.1
ssc-miR-9842-5p	0	0	0	0	0	0	0	0
ssc-miR-9843-3p	18.63	7.48	10.84	11.57	79.44	56.54	51.03	73.82
ssc-miR-9844-3p	0	0	0	0	0	0	0	0
ssc-miR-9846-3p	0	0	0	0	0	0	0	0
ssc-miR-9848-3p	0	0.07	0	0	0.06	0	0	0
ssc-miR-9849-5p	0	0	0	0	0	0	0	0
ssc-miR-9850-5p	0	0	0	0	0	0	0	0
ssc-miR-9851-3p	1.83	0.9	2.01	1.82	41.21	14.33	9.23	40.39
ssc-miR-9852-3p	0	0	0	0	0	0	0	0
ssc-miR-9853-5p	0	0	0	0	0	0	0	0
ssc-miR-9854-5p	0	0	0	0	0	0	0	0
ssc-miR-9855-5p	0	0	0	0	0	0	0	0
ssc-miR-9857-5p	0	0	0	0	0	0	0	0
ssc-miR-9858-5p	1.07	0.3	0.43	0.59	7.63	5.72	3.52	8.65
ssc-miR-9859-3p	0	0	0	0	0	0	0	0
ssc-miR-9860-5p	2.97	1.5	1.65	0.65	2.11	1.23	1.41	2.66
ssc-miR-9861-5p	0	0	0	0	0	0	0	0
ssc-miR-99a	1026	550.35	708.7	966.68	657.7	402.26	396.3	928.53
ssc-miR-99b	497.2	254.18	338.8	318.67	1013	1086.6	932.6	1479.8

Excel S2 Details of novel miRNAs-count

ID	miRNA_Seq	D70F1 RPM	D70F2 RPM	D70M1 RPM	D70M2 RPM	P70F2 RPM	P70F1 RPM	P70M1 RPM	P70M2 RPM
Novel_0	aaacccuggagcuuucacacacua	1.33	0	0	0	0	0	0	0.89
Novel_1	aaagcaggauuuagacuacaau	0	0	0	0	0.93	0	0	1.02
Novel_2	aaaugcuggcgcuuucacacacuc	1.07	0	0	0	0	0	0	0
Novel_3	aacccuggagcuuucacacacu	0	0	0	1.82	0	0	0	0
Novel_4	aaccugguugucauugucuuga	1.64	0	2.44	1.3	1.24	0	0	0
Novel_5	aagaggaacgugacugagagac	0	0	0	0	0	1.09	0	0
Novel_6	aaggcgggagaagucccg	0	0	0	0	0	0	0	1.63
Novel_7	aaggcgggagaagucccggc	0	0	0	0	0	0	0.63	0
Novel_8	aauagccaguuacucgucagc	0	0	0	0	0	0	0	1.98
Novel_9	aauuggggcugucaggcc	0	0	0	0	0	0	0	0.14
Novel_10	acaaggcuagaggucgaaucaga	0	0	0	0	0.99	0	0	1.02
Novel_11	acggaacgagagagaga	0	0	0	0	0	0	0	0.41
Novel_12	acucugccuucugucccaggcug	0	0	0	0	0	0	0	0.54
Novel_13	agccaggauuguggguggcagcca	1.2	0	0	0	0	0	0	0
Novel_14	agcggccagaacugccucugacu	0	0	0.65	0	1.24	0.65	0.85	0
Novel_15	agcugagcgccggacgcgcgcgg	0	0	0	0	0.19	0	0	0
Novel_16	aggacgggaagagaggag	0	0.67	0	0	0	0	0	0
Novel_17	aggacgggaagagaggagg	0.57	0	0	1.69	0	70.07	0	65.65
Novel_18	aggacgggaagagaggaggg	0	0	0	0	45.62	0	18.82	0
Novel_19	aggauggacaguaauuc	0	0	0	0.98	0	0	0	0
Novel_20	aggcagcggagggacucccug	0	0	0	0	0	0	0.56	0
Novel_21	aggcccuuacauagucagacuc	0.38	0	0.43	0	0	0	0	0.54
Novel_22	agggagagaacaccaucugaguggu	0	0	16.37	0	0	0	0	0
Novel_23	aguuagaagaauaaagaugcu	0.51	0.15	0	0	0	0	0	0
Novel_24	aguucgagccgacgggcugc	0	0	0	0	0	1.23	0	0
Novel_25	auaccgggugcuguaggcuu	0	0	2065.05	0	0	0	0	0
Novel_26	auggacuaacugugguauugg	0	0	0	0	0	0	0	0.48
Novel_27	auggacuaacugugguauuggga	0	0	0	0	0	0	0.49	0
Novel_28	auucaggaccuggucuguggauu	0	0.3	0	0	0	0	0	0
Novel_29	auucuggagcucauggucuucu	0.32	0	0	0	0	0	0	0
Novel_30	auucuggagcucauggucuucugc	0	0	0	0	0	0	0	0.61
Novel_31	auugagaacacugacauaac	0	0	0	0	0	0.14	0	0
Novel_32	caauuuagguuaaugacugucu	1.2	0	0	0	0	0	0.63	1.43
Novel_33	caccccagacccaauggcaaga	0.32	0	0	0	0	0	0.14	0
Novel_34	cagaggccagagggcaggacgcu	2.02	0.9	0	0.65	0	0	0	0
Novel_35	caggcagcagggaggucgccugc	0.19	0	0	0	0	0.36	0	0
Novel_36	caguagguacagaacagccuucu	0.13	0	0	0	0	0	0	0
Novel_37	cagucgggacgcuuccuucuu	0	0	0	0	0	0	0	0.27
Novel_38	cagucgggacgcuuccuucuuga	0	0	0	0	0.37	0	0	0
Novel_39	cagugccaggugggggaguuugac	99.73	0	206.53	0	165.46	0	0	0
Novel_40	caucccgauugaaugcaguggauac	0	0	0	0	1.92	0	0	0
Novel_41	cauugugccaugaugca	0	0	0	0	0	0	0	11.78
Novel_42	ccauugcaccacaaugca	0.76	0	0	0	22.03	37.35	0	35.75
Novel_43	ccauugcgccacaaugca	2.21	3.52	1.94	5.79	125.18	240.26	36.86	290.6
Novel_44	ccauugcgccucaaugca	0	0	0	0	0	1.09	0	2.66
Novel_45	ccauugugccacaaugca	0	0	0	2.99	0	0	0	54.55
Novel_46	cccaucucuaaguccugugga	0	0	0	0	0.62	0	0	0
Novel_47	cccccgcgagggacacccccc	0	0	0	0	0	0	0	0.34
Novel_48	ccccgacaccauggcccuggc	0.19	0	0	0	0	0.43	0	0
Novel_49	ccccgccuccucucgcc	2.08	0	6.82	0	0	0	0	5.45
Novel_50	ccccugcagugucugugccacc	0	0	0	0	2.67	1.09	1.76	5.45
Novel_51	ccccugggcagcggggcc	0	0	0	0	0	0.58	0	2.32
Novel_52	ccccuggggguccacaggggg	0	0	0	0	0	0	0	0.2
Novel_53	cccgcccgggagccggggcugc	0	0	0	0	0	0.43	0	0
Novel_54	cccgccuccucucgccgcc	0	0	0	0	0	0	1.27	0
Novel_55	cccgcgcgcgcgcgcgcgcc	0	0	0	0	0.68	0	0	0
Novel_56	cccggguccguguggcccucga	0	0	0	0	0	0.22	0	0
Novel_57	cccgugggugucgaccagcugucc	0	0	0	0	0	0	0.99	0
Novel_58	cccucaacacccccaagguuca	0	0	0	0	0	0	0.99	0
Novel_59	cccugucucacugucuccuucc	0	0	0	0	1.24	0	0.21	0.95
Novel_60	ccgcccuuccucaacacc	0	0	0	0	0	0	0	1.43
Novel_61	ccgcggcccuuucuuccauucuugu	0.19	0	0.22	0	0	0.65	0	0
Novel_62	ccggaaguggaaguaagcgcagg	0	0	0	0	0	0	0	0.54
Novel_63	ccgguucccgagggcccgugu	0	0	0	0	0.25	0	0	0.2
Novel_64	ccgugggugucgaccagcugucc	0	0	0	0	0	0.94	0	0
Novel_65	ccucaccgcgccggcuucucuc	0	0	0	0.46	0	0	0	0
Novel_66	ccugggcccugcugcuuugc	0	0	0	0	0	0	3.52	0
Novel_67	ccuguuccaauggcucuucc	0	0	0	0	0	0	0	1.23
Novel_68	ccuugacucacacaggccc	0	0	0	0	0	0	0	0.2
Novel_69	cggcugcaccaggacaggaacuc	0	0	0	0	0.43	0	0	0
Novel_70	cuaacucccguguacugcacu	0.95	0	0	1.17	4.16	1.3	1.2	4.36

Novel_71	cuauagauucugauucaguag	0	0	0.14	0	0	0	0	0
Novel_72	cucacucaguggguuggggaugugg	0	0.15	0	0	0	0	0	0
Novel_73	cucaggacccaagugcccgcauu	0	0	0	0	0.87	0	0	0
Novel_74	cuccccgacaccauggcccuggc	0	0	0	0	0	0	0.21	0
Novel_75	cucccuagcuggcuaaugg	0	0	0	0	0	1.16	0	0
Novel_76	cuccugcaucgaaagugaucgug	0.63	0	0	0	0	0	0	0
Novel_77	cuccuucaggaagcucugucgg	0	0	0	0	0.25	0	0.14	0
Novel_78	cucgcaguuguuggcagcccuggc	0	0	0	0	0	0	0.35	0
Novel_79	cucuccucccgucuucc	0	0	0	0	0	2.61	0	0
Novel_80	cucuuggcugucugagcauu	0	0	0	0	0	0	0	1.43
Novel_81	cugagacaauuccauucagugu	1.52	0.3	0.93	1.5	1.12	0.43	0.49	1.09
Novel_82	cugauggccucugucccccaga	0	0	0	0	1.06	0	0.63	1.09
Novel_83	cugcauucaaucacaaugca	0	0	0	0	11.17	14.55	0	24.99
Novel_84	cugcccaccugggugagaagga	0	0	0	0	0	0	0	0.61
Novel_85	cugccguugggucuggggugu	0	0	0	0	0	0	0	0.48
Novel_86	cugccguugggucuggggugug	0	0	0	0	0	0.43	0	0
Novel_87	cugccugagucugggaaugc	0	0	0	0	0	0	0	2.45
Novel_88	cugcuggaggacgcccggugu	0	0	0	0	0.81	0	0	0.75
Novel_89	cuguaccaggaugccagcauaa	0	0	0	0	0.87	0	0	0
Novel_90	cugugugguucugaggcu	0	0	0	0	0.5	0	0	0
Novel_91	cuguugccacuaaccucaacc	0	0	0	0	22.84	8.18	8.67	28.33
Novel_92	cuguugccacuaaccucaaccu	7.52	3.22	2.01	3.9	0	0	0	0
Novel_93	cuuccugucuccuccuucccagu	0	0	0	0	0.5	0	0	0
Novel_94	cuuucuguacccaccugcuagu	0	0	0	0	0	0	0	0.27
Novel_95	gagcuucugcgcaucccggaga	0.44	0	0	0	0	0	0	0
Novel_96	gaugggaacucccugagccauu	0	0	0	0	0.74	0	0	0.89
Novel_97	gcaagucagcauggccug	0	0	0	0	0	0	0	0.54
Novel_98	gcaauuuagguuaaugacugucu	0	1.05	0.72	0.85	0.74	0	0	0
Novel_99	gcaccuuuauucuucuaacug	0	0	0.57	0.39	0	0	0	0.34
Novel_100	gcaggcugcugguuguc	0	0	0	0	0.37	0	0	0
Novel_101	gccgaagaccugaagggg	0	0	0	0	0	0	0	0.48
Novel_102	gccggaugcagacaguggug	0	0	0	0	0.12	0	0	0
Novel_103	gccggcggcggcggcgac	0	0	0	1.56	0	0	0	0
Novel_104	gcgaagacugagcgguugcgg	0	0	0	0.65	0	0	0	0
Novel_105	gcucgcgggcuggccuaccugc	0	0	0	0	0.5	0	0	0
Novel_106	gcugggucggucggguugg	0	0	0	0	0	0.65	0	0
Novel_107	ggagagaacaccgucugaguggu	0	0	0	0	0	0	0	23.77
Novel_108	ggauaaagaagcagaugcugugac	0	0	0	0	0	0.72	0	0
Novel_109	ggcccuuacauagucagacuc	0	0	0	0	0.43	0	0	0
Novel_110	gggugggggucugggggggag	0	1.12	0	0	0	0	0	0
Novel_111	gguguaugugcuuggcug	0	0	0	0	0	6.01	0.78	0
Novel_112	gguucucaccucgcugguguccc	0	0	0	0	0	0	0.7	0
Novel_113	guggauucacucaacuuggguu	0	0	0	0.72	2.05	0.94	0	1.23
Novel_114	guggauucacucaacuuggguuu	0	0	0	0	0	0	0.92	0
Novel_115	guucucaccucgcugguguccc	0	0	0	0	1.18	0.65	0	1.63
Novel_116	uaacagucuccagucacggccac	0	0	0	0	0	0	0	0.2
Novel_117	uaaggaucugaccuggucacu	0	0	0	0	0	0	0	0.61
Novel_118	uaauacugccugguaaugaugac	79.39	35.55	0	56.76	337.49	0	180.02	333.16
Novel_119	uacaugugcaagaugcagaaggu	0	0	0	0	0	0	0	1.36
Novel_120	uaccauggcacuggcagagccc	0	0	0	0	0.43	0	0	0
Novel_121	uacugcaaagugauugaggagc	0.69	0	0.65	0	0	0	0	0
Novel_122	uagcucaguggcagagcauuugacu	0	4.12	0	0	0	0	0	0
Novel_123	uaggggucagaucagagcuguaga	0	0	0	0	0	0.29	0	0
Novel_124	uagucucccauugucaacagucu	0	0	0	0	0.12	0	0	0
Novel_125	uauccagacaggugcuguucuc	0	0	0	0	0.68	0	0	0
Novel_126	uauccaggcaggugcuguucucu	0	0	0	0	0.5	0	0	0
Novel_127	uaugggucaucucccuugaacu	0.13	0	0	0	0	0	0	0
Novel_128	ucaggacccaagugcccgcauugg	0.44	0.6	0	0	0	0	1.41	1.5
Novel_129	uccaacguggauaccccggga	0	0	0	0	0	0	0	0.75
Novel_130	uccagaaccucauggugcugagg	0	0	0	0	0	0	0.14	0
Novel_131	uccaggagccggguccaggga	0	0	0	0	0	0.58	0.49	0.34
Novel_132	ucccaucugggucgcca	0	0	0	0.52	0	1.59	0	0.82
Novel_133	ucccuguccuccaggagcucau	0	0	4.81	0	0	0	0	0
Novel_134	uccgaggucuugugagauggac	0.51	0	0	0	0	0	0	0
Novel_135	uccguaauuacucauguaccca	0	0	0.57	0	0	0	0	0
Novel_136	uccuagucugggugcaaacaguu	0	0	0	1.24	0	0	0	0.89
Novel_137	ucggcagcugcuuggcguccga	0.51	0	0	0	0	0	0	0.61
Novel_138	ucgugcacagauguggucucg	0	0	0	0	0	0	0	0.34
Novel_139	ucuccuggcuggcucgcca	0.69	2.17	1.44	10.27	2.23	9.7	1.69	2.86
Novel_140	ucucucaauugucucaacagcc	0	0	0.22	0	0	0.51	0.63	0
Novel_141	ucucucuucucucuguggg	0	0	0	0	0	0	0	0.2
Novel_142	ucuggcuguggucuagacuggc	0	0	0	0	0	0	0	0.68
Novel_143	ucuggcugugguguagaccgu	0	0.9	0	0	0	0	0	0

Novel_144	ucuggcuguggugguagaccguc	3.79	0	2.8	1.89	16.69	11.36	6.77	18.59
Novel_145	ucugggcacuucagcugguggu	0	0	0	0	0.56	0	0.28	0.75
Novel_146	ucugggcagagguagacagggugg	0	0	0	0	0.25	0	0	0
Novel_147	ugaauggcgccuuucugaguaga	0	0	0.72	0	0	0	0	0
Novel_148	ugagugugugugugugaguguau	0.44	0	0	0	0	0	0	0
Novel_149	ugcccuugugccugggugg	0	0	0	0	0	0	0	0.34
Novel_150	ugcgggugagacggaagaacac	0	0	0	0	0	0	0.42	0.54
Novel_151	ugcucugugaugaaccc	0	0	0	0	0	0	0	9.6
Novel_152	uggauuguucuccaaccuggcucu	0	0.3	0	0	0	0	0.42	0
Novel_153	uggccagcuguuuccucucuuu	0	0	0	0	1.06	0	0	0.75
Novel_154	uggcuccacaccacagacuguc	0	0	0	0	0.5	0	0	0.75
Novel_155	uggcuuguacucuaggug	0	0	0	0	0	0	0	0.82
Novel_156	uggucaaggccugucgccgcugu	0	0	0	0	0.5	0	0	0
Novel_157	uguaguccuggaguucccaucg	0	0	0	0	0	0	0	0.14
Novel_158	ugugacgugacaugggcaagc	0	0	0	0	0	0	0	0.61
Novel_159	uguggcuguggcguaugccaa	0	0	0	0	0.74	0	0	0
Novel_160	uguggcuguggcugugacccgc	0	0	0	0	0.87	0	0	0
Novel_161	ugugucugugacaucugcuggca	2.72	1.57	1.87	1.04	8.32	2.75	6.84	7.49
Novel_162	ugugugggcuaagugguagauuu	0	0	0	0.33	0	0	0	0
Novel_163	uguguggguucugaggcu	0	0	0	0	0	0.8	0	0
Novel_164	ugugugugagcaucugcguggac	0	0	0	0	0	0.22	0	0
Novel_165	uguucccucuggugacuucugga	2.59	0	0	0	0	0	0	0
Novel_166	uguuuagcguguggcaugggau	0	0	0	0	0	0	0	0.2
Novel_167	uuauuaaauaaugccuugaggu	0.19	0	0.29	0	0	0	0	0.27
Novel_168	uuauucaguggcccucaaucccu	0.32	0	0	0	0.56	0	0	0.75
Novel_169	uucauggauugucuucugggua	0	0	0	0	0.43	0	0	0
Novel_170	uucccagaacgcaagacucuag	0	0	0	0	0	0	0	0.68
Novel_171	uucccuccugcugguacuccc	0	0	0	0	0	0	0	0.48
Novel_172	uugacaacagcucucaacuggu	0	0	0	0	1.37	0	0	1.16
Novel_173	uugagacaauugagagaugauu	0	0	0	0	0.62	0	0	0.34
Novel_174	uugcagugugcugaaaccucgg	0	0	0	0	0.81	0	0	0
Novel_175	uugcagugugcugaaaccucggc	0	0	0.57	0	0	0	0.63	0.89
Novel_176	uuggcggggguacccugaacc	0	0	0	0	0.25	0	0	0
Novel_177	uuggcggggguacccugaacca	0	0	0	0	0	0	0	0.54
Novel_178	uuggcucugcgaggucggcuca	18.25	8.53	13.71	12.35	131.82	55.52	63.72	171.28
Novel_179	uuggggugaagaauauacaggg	0	0	0	0	0.19	0	0	0
Novel_180	uugguuuguuuggguuuguu	0	0	0.65	0	0	0	0	0
Novel_181	uuguucacccacuaauagg	0	0	0	0	0	0.65	0	0
Novel_182	uuuacguccuuucaccuaguuu	6.44	0	2.58	3.77	6.45	3.26	5.78	5.04
Novel_183	uuugcagugugcugaaaccucgg	1.01	0	0	0	0	0	0	0
Novel_184	uuugguuuguuuggguuuguu	0	0	0	0	0.19	0	0	0
Novel_185	uuugucuuaaucucugugguuu	0.19	0	0	0	0	0	0	0
Novel_186	uuuguuggcuccucugaagugag	0.13	0	0	0	0	0	0	0.34

Excel S3 Details of differentially expressed miRNAs

miRNA	log2FoldChang	padj	Up/Down	Significant
ssc-miR-192	-1.998253206	7.39E-13	down	yes
ssc-miR-26a	-1.487391203	4.28E-07	down	yes
ssc-miR-30a-5p	-1.103577495	0.000338	down	yes
ssc-miR-451	2.375383431	6.13E-05	up	yes
ssc-miR-194a	-1.930917256	3.42E-09	down	yes
ssc-miR-199b-3p	-2.142890843	6.47E-11	down	yes
ssc-miR-199a-3p	-2.142892276	6.47E-11	down	yes
ssc-miR-144	3.863486039	7.56E-11	up	yes
ssc-miR-21	-2.524439725	1.65E-09	down	yes
ssc-let-7f	-3.053927101	1.45E-21	down	yes
ssc-miR-27b-3p	-1.368286495	6.13E-05	down	yes
ssc-miR-125b	-1.310770152	4.72E-05	down	yes
ssc-miR-222	1.819554882	1.01E-07	up	yes
ssc-miR-10a-5p	-2.144180855	8.58E-05	down	yes
ssc-miR-29a	-1.905086142	7.97E-09	down	yes
ssc-miR-199a-5p	-1.942890783	2.74E-07	down	yes
ssc-miR-107	1.029632216	0.013971192	up	yes
ssc-miR-486	3.22744352	1.48E-19	up	yes
ssc-miR-17-5p	1.62991303	1.62E-05	up	yes
ssc-miR-20a	1.043083356	0.029204187	up	yes
ssc-let-7g	-2.899737098	8.87E-18	down	yes
ssc-miR-184	6.891436231	2.91E-41	up	yes
ssc-miR-532-5p	2.521366845	2.33E-13	up	yes
ssc-miR-127	2.834769888	1.97E-10	up	yes
ssc-miR-181b	-1.005459631	0.006162008	down	yes
ssc-let-7c	-2.129940567	2.85E-10	down	yes
ssc-miR-500	3.023473103	5.16E-13	up	yes
ssc-miR-130b	1.603270705	9.97E-07	up	yes
ssc-miR-99a	-1.313471115	0.000134817	down	yes
ssc-miR-378	-1.392583567	1.82E-05	down	yes
ssc-miR-542-3p	2.011699544	2.00E-09	up	yes
ssc-miR-874	1.100594728	0.00164935	up	yes
ssc-miR-199b-5p	-1.850608097	6.69E-07	down	yes
ssc-miR-423-5p	-1.688449086	0.013815883	down	yes
ssc-miR-497	-1.7399555	2.07E-07	down	yes
ssc-miR-18a	3.071754405	6.43E-17	up	yes
ssc-miR-296-3p	2.94211386	1.37E-12	up	yes
ssc-miR-421-3p	1.888398842	6.41E-09	up	yes
ssc-miR-98	-1.817292686	7.55E-08	down	yes
ssc-miR-24-3p	-1.217315021	0.002720706	down	yes
ssc-miR-23b	-1.569356813	4.35E-06	down	yes
ssc-miR-27a	-2.40746667	5.70E-13	down	yes
ssc-miR-150	-1.641310988	8.77E-05	down	yes

ssc-miR-744	2.078444927	6.90E-10	up	yes
ssc-miR-29c	-1.293615183	4.20E-05	down	yes
ssc-let-7d-5p	-3.127635436	1.63E-19	down	yes
ssc-miR-505	-1.191426821	0.001692611	down	yes
ssc-miR-29b	-1.051410195	0.024768544	down	yes
ssc-miR-542-5p	1.920954102	2.22E-08	up	yes
ssc-miR-23a	-2.024309646	2.95E-09	down	yes
ssc-miR-324	1.416543384	7.82E-05	up	yes
ssc-miR-17-3p	1.081339751	0.002041994	up	yes
ssc-miR-429	1.577866336	1.39E-05	up	yes
ssc-miR-32	-1.44539232	2.72E-05	down	yes
ssc-miR-1296-5p	1.672044415	3.41E-06	up	yes
ssc-miR-183	3.241044421	1.04E-17	up	yes
ssc-miR-532-3p	3.017869875	1.43E-15	up	yes
ssc-miR-361-3p	-1.648920473	1.18E-06	down	yes
ssc-miR-9843-3p	1.679820887	1.11E-05	up	yes
ssc-miR-214	-2.120174782	8.30E-10	down	yes
ssc-miR-362	2.040736454	0.000328417	up	yes
ssc-miR-195	-2.642992152	6.52E-11	down	yes
ssc-miR-146a-5p	-3.162839461	1.03E-19	down	yes
ssc-miR-582	1.044761982	0.013815883	up	yes
ssc-miR-376a-5p	5.031786538	4.64E-32	up	yes
ssc-miR-145-3p	-2.219142812	2.56E-10	down	yes
ssc-miR-432-5p	3.333247581	1.01E-16	up	yes
ssc-miR-378b-3p	-1.597919807	1.44E-05	down	yes
ssc-miR-9851-3p	3.027102792	8.14E-07	up	yes
ssc-miR-210	2.482944956	4.80E-09	up	yes
ssc-miR-1306-5p	1.339258171	0.001269035	up	yes
ssc-miR-215	-3.009587905	0.000230299	down	yes
ssc-miR-205	2.030628333	0.001648414	up	yes
ssc-miR-7139-5p	1.660616943	0.00023451	up	yes
ssc-miR-18b	2.076352429	7.67E-05	up	yes
ssc-let-7d-3p	-1.335000532	0.000396025	down	yes
ssc-miR-296-5p	1.431168097	0.000787581	up	yes
ssc-miR-10a-3p	-1.601210282	1.07E-05	down	yes
ssc-miR-24-1-5p	-1.337740348	0.007129064	down	yes
ssc-miR-331-5p	-1.080111804	0.006595507	down	yes
ssc-miR-20b	1.235280091	0.002915705	up	yes
ssc-miR-137	2.678989441	0.006595507	up	yes
ssc-miR-217	4.261624479	5.20E-16	up	yes
ssc-miR-193a-3p	-3.306255054	2.60E-19	down	yes
ssc-miR-216	3.81149734	2.00E-13	up	yes
ssc-miR-95	-2.739504345	7.87E-13	down	yes
ssc-miR-493-5p	3.124858999	8.81E-09	up	yes
ssc-miR-551a	2.532292456	2.74E-07	up	yes

ssc-miR-7144-5p	1.344165199	0.007129064	up	yes
ssc-miR-9858-5p	2.683600302	2.17E-07	up	yes
ssc-miR-1306-3p	1.407648628	0.002267962	up	yes
ssc-miR-664-3p	-1.154112232	0.008756098	down	yes
ssc-miR-493-3p	3.536175371	3.44E-09	up	yes
ssc-miR-191	-2.383052283	0.00272104	down	yes
ssc-miR-450b-5p	1.976969764	0.000303992	up	yes
ssc-miR-421-5p	1.714059259	0.002990457	up	yes
ssc-miR-106a	1.808869819	0.00157839	up	yes
ssc-miR-2411	1.349341111	0.026903184	up	yes
ssc-miR-381	3.521908843	2.08E-07	up	yes
ssc-miR-7139-3p	1.234555713	0.036151582	up	yes
ssc-miR-24-2-5p	-1.577083957	0.000458773	down	yes
ssc-miR-9810-3p	2.574025681	7.67E-05	up	yes
ssc-miR-194b-5p	-2.283115051	0.002620674	down	yes
ssc-miR-202-5p	3.017580722	0.022480438	up	yes
ssc-miR-7138-3p	2.175360102	0.010426176	up	yes
ssc-miR-450c-5p	2.319595407	0.008756098	up	yes
ssc-miR-1249	-1.730686893	0.003288524	down	yes
ssc-miR-7857-3p	3.61884516	0.001269035	up	yes
ssc-miR-124a	3.903910885	0.049755731	up	yes
ssc-miR-376c	Inf	0.005829536	up	yes
ssc-miR-432-3p	3.077817942	0.026903184	up	yes
ssc-miR-145-5p	-2.004484119	0.005087638	down	yes
ssc-miR-155-3p	-3.49711849	0.00624047	down	yes
ssc-miR-193a-5p	-3.972615994	2.59E-09	down	yes
ssc-miR-7135-3p	-2.507475142	0.005589947	down	yes
ssc-miR-365-5p	-2.619235452	0.009842366	down	yes
